# MAPPIN: a method for annotating, predicting pathogenicity and mode of inheritance for nonsynonymous variants

**DOI:** 10.1093/nar/gkx730

**Published:** 2017-08-25

**Authors:** Nehal Gosalia, Aris N. Economides, Frederick E. Dewey, Suganthi Balasubramanian

**Affiliations:** 1 Regeneron Genetics Center, Tarrytown, NY 10591, USA; 2 Regeneron Pharmaceuticals, Tarrytown, NY 10591, USA

## Abstract

Nonsynonymous single nucleotide variants (nsSNVs) constitute about 50% of known disease-causing mutations and understanding their functional impact is an area of active research. Existing algorithms predict pathogenicity of nsSNVs; however, they are unable to differentiate heterozygous, dominant disease-causing variants from heterozygous carrier variants that lead to disease only in the homozygous state. Here, we present MAPPIN (**M**ethod for **A**nnotating, **P**redicting **P**athogenicity, and mode of **I**nheritance for **N**onsynonymous variants), a prediction method which utilizes a random forest algorithm to distinguish between nsSNVs with dominant, recessive, and benign effects. We apply MAPPIN to a set of Mendelian disease-causing mutations and accurately predict pathogenicity for all mutations. Furthermore, MAPPIN predicts mode of inheritance correctly for 70.3% of nsSNVs. MAPPIN also correctly predicts pathogenicity for 87.3% of mutations from the Deciphering Developmental Disorders Study with a 78.5% accuracy for mode of inheritance. When tested on a larger collection of mutations from the Human Gene Mutation Database, MAPPIN is able to significantly discriminate between mutations in known dominant and recessive genes. Finally, we demonstrate that MAPPIN outperforms CADD and Eigen in predicting disease inheritance modes for all validation datasets. To our knowledge, MAPPIN is the first nsSNV pathogenicity prediction algorithm that provides mode of inheritance predictions, adding another layer of information for variant prioritization.

## INTRODUCTION

Whole-exome sequencing (WES) is increasingly being used to identify causal mutations leading to a disease or phenotype. Nonsynonymous single nucleotide variants (nsSNVs) comprise a significant portion of the observed variation with an average exome containing 10,500–13,500 nsSNVs (1–4). A few studies have demonstrated that this number is lower than expected, suggesting that nsSNVs are subject to negative selection due to deleterious effects on gene function (5,6). Moreover, according to the Human Gene Mutation Database (HGMD), nsSNVs constitute ∼45% of disease-causing mutations across 5,700 genes (7). Therefore, a critical challenge in the interpretation of WES data is the differentiation of potential disease-causing mutations from those that are either tolerated or benign. An important step in the identification of deleterious variants is their prioritization using *in silico* prediction programs that are able to distinguish pathogenic from benign variants (8). Pathogenic mutations tend to be detected in evolutionarily conserved regions and are enriched within important functional domains (9,10). Therefore, many widely used nsSNV prediction methods utilize conservation and structure-based features as the main criteria for scoring variant pathogenicity. These methods use some shared and some distinct features to provide a score that indicates the likelihood of a variant being deleterious. Some commonly used algorithms include SIFT (11), PolyPhen2 (12), MutationTaster2 (13), CADD (14), and LRT (15). Several studies show that combining various algorithms and their unique features increases prediction accuracy. For example, both the CONDEL and the Logit model combine scores from five prediction programs to improve prediction accuracy (16,17). CADD integrates scores from SIFT, PolyPhen and features such as GERP scores, DNase I hypersensitivity sites and Grantham scores to predict deleteriousness for SNVs and small insertions or deletions (14). Improvements obtained by combining scores from multiple methods indicate that there is scope for further development in the field of nsSNV prediction algorithms.

An issue with existing algorithms is the lack of information that allows discrimination between dominant and recessive disease-causing mutations. Furney *et al.*, identified differences in sequence conservation, gene essentiality, and paralogy between dominant and recessive genes (18). This suggested that certain features could be incorporated into prediction algorithms to distinguish between dominant and recessive disease-causing variants. To determine if existing algorithms could differentiate dominant and recessive variants, Li *et al.* evaluated several well-known and commonly used algorithms individually and in combination, on a known set of dominant and recessive disease-causing mutations (17). The algorithms tested by Li *et al.* included PhyloP, MutationTaster, LRT, PolyPhen2, SIFT, and a combination of all five methods. Although the prediction scores for the known dominant and recessive mutations were different and statistically significant for a few methods, for most algorithms, the difference between the dominant and recessive scores was very small (17). Moreover, a plot of the true positive versus the false positive rate for the dominant and recessive variants showed an area under the curve (AUC) of ∼0.5 for 3-fold cross-validation. This suggests that the existing algorithms do not include the features necessary to differentiate between autosomal dominant and recessive disease-causing variants.

Methods that can predict the impact of variants and their mode of inheritance are valuable in both Mendelian family-based analyses and population genetics approaches. Typically, identification of causal variants in exome sequencing studies is performed by identifying variants that segregate with affected probands. When the inheritance mode is known, it is easier to design a filtering strategy to identify causal variants from a list of candidate variants that segregate with probands. However, often there is not enough information or there are too few family members or families to identify alleles segregating with affected individuals requiring the use of multiple models with different modes of inheritance (19,20). In such cases, variants can be prioritized based on a combination of predicted pathogenicity and mode of inheritance. Additionally, although discrete filtering methods work well for recessive disorders, they are less useful for dominant diseases as an individual can harbor 10,000–12,000 nsSNVs (21). Moreover, studies suggest that 50-fold more genes are likely to harbor heterozygous versus homozygous protein-altering variants, markedly increasing the complexity of the analyses. This is reflected in the greater success of WES studies in identifying causal genes for recessive diseases compared to dominant diseases (22). Thus, methods to identify candidate dominant heterozygous mutations and distinguishing them from carrier variants that lead to disease only in the homozygous state and also from benign variants are essential. Therefore, we developed MAPPIN (Method for Annotating, Predicting Pathogenicity and mode of Inheritance for Nonsynonymous variants), a method to predict pathogenicity and mode of inheritance for nsSNVs.

**Figure 1.**
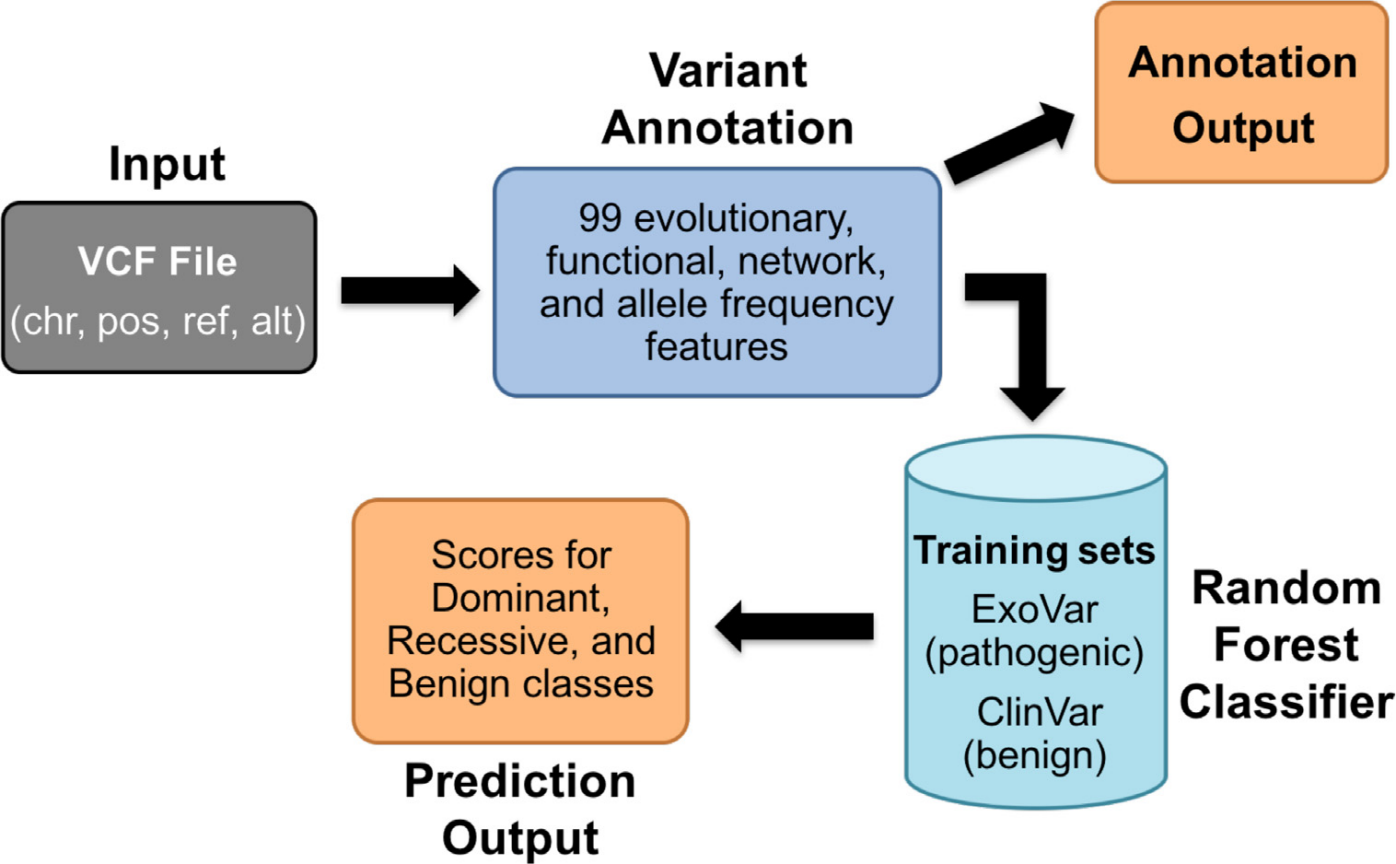
MAPPIN workflow. Figure depicting the workflow for MAPPIN from user input of a VCF file to outputs at two stages: one with variant annotations and a second with predictions derived from a random forest classifier.

MAPPIN adopts an approach similar to ALoFT (Annotation of Loss-of-Function Transcripts), which predicts pathogenicity and mode of inheritance for protein-truncating variants including premature stop and frameshift indel mutations (http://aloft.gersteinlab.org/, http://biorxiv.org/content/early/2017/02/07/106468). MAPPIN initially annotates nsSNVs with 99 features encompassing conservation metrics, protein domain and post-translational modifications, biological networks and allele frequency information (Supplementary Table S1). Subsequently, MAPPIN classifies variants into three classes: dominant (disease-causing as heterozygotes), recessive (disease-causing when homozygous or compound heterozygous) and benign using a random forest classifier trained on known benign and deleterious variants (Figure 1). To the best of our knowledge, this is the first algorithm that classifies all possible nsSNVs into benign, dominant and recessive classes providing three probability scores as opposed to the single pathogenicity score provided by existing algorithms. Finally, and importantly, MAPPIN is the first nsSNV prediction program that is able to distinguish dominantly-acting and recessively-acting variants from each other.

## METHODS

### Pipeline for annotating variants

The annotation pipeline for MAPPIN can directly use VCF files or tab delimited files, which at a minimum must include the chromosome (#CHROM), position (POS), reference (REF) and alternate alleles (ALT). Initially, a sequence-based annotation is performed using the Variant Annotation Tool (VAT) (23). Next, the variants are annotated with many different features that provide additional information and are described in further detail below.

#### Evolutionary features

Genomic evolutionary rate profiling (GERP) scores and presence of the variant within a GERP element are provided as measures of constraint (24). Ratios of nonsynonymous/synonymous substitutions (dN/dS) comparing human to macaque and human to mouse homologs obtained from Ensembl release 75 are also included in the output (25).

#### Allele frequency

MAPPIN annotates variants with allele frequency information from different reference datasets. The datasets used are: 1000 Genomes Project (26), ESP6500 from the Exome Variant Server (27) and the ExAC dataset including TCGA samples (28). Additionally, MAPPIN also utilizes the pLI score, which is a measure of constraint indicating the probability that a gene is intolerant to loss-of-function mutations and thus measures haploinsufficiency. pLI is derived from ExAC data from the following file: fordist_cleaned_exac_nonTCGA_z_pli_rec_null_data.txt (29).

#### Functional features

The location of the variants within specific protein domains of a gene is annotated. These include PFAM, SMART, SCOP super family, signal peptide and transmembrane helix domains obtained from Ensembl release 75 (25). Additional protein features include post-translational modifications such as phosphorylation, methylation, acetylation, O-β-linked N-acetylglucosamine, sumoylation, and ubiquitination from PhosphoSite Plus (30). Disordered residues based on predictions from DISOPRED2 are also annotated because long (>30 residues) disordered sequences are present in up to third of eukaryotic proteins and have been shown to be important for transcriptional regulation and signaling (31). Validated miRNA binding sites are included from miRWalk (32). Lastly, splice score predictions for all SNPs that occur within splicing consensus regions that may alter splicing are derived from dbNSFP and annotated by MAPPIN (33). The splice scores in dbNSFP are generated using ensemble learning methods, which combine multiple existing algorithms such as the Position Weight Matrix model, MaxEntScan, and other conservation-based features to predict the functional effect of a splice variant.

#### Network features

Human protein–protein interaction networks were downloaded from BIOGRID version 3.4.128 (34). Dominant and recessive disease-causing genes were obtained from curated lists from Online Inheritance In Man (OMIM) (35–37). The shortest paths to dominant and recessive disease-causing genes are also calculated and included in the output. Additionally, gene centrality scores within various networks such as metabolic, genetic, and signaling obtained from Khurana *et al.* are annotated (38). Tissue expression data from 25 tissues through the Genotype-Tissue Expression project are included (39). Transcript-based expression levels are used to calculate the mean expression value across all individuals for each tissue. An entropy score is also calculated for each gene as a measure of overall tissue specificity of expression using the Shannon entropy method (40,41).

#### Gene and transcript features

Gene and transcript based features include: Ensembl transcript ID, length of the transcript, whether the transcript isoform is the longest, and if the variant affects all (full) or only some (partial) transcript isoforms. Additionally, to account for mismapping errors and gene function compensation, the program includes annotations for whether the gene has segmentally duplicated regions, pseudogenes (42), and paralogs (25).

Other features added are synonymous and nonsynonymous SNP density based on the 1000 Genomes Project, GERP scores for synonymous and nonsynonymous SNPs including percentage of each class of polymorphisms in GERP elements, percent of transcript composed of GERP elements and average heterozygosity of synonymous and nonsynonymous SNPs in phase 1 of the 1000 Genomes data subdivided by ethnicity. Average heterozygosity is calculated as ${2pq\over l}$, where p is minor allele frequency, q is reference allele frequency, and l is coding transcript length.

### MAPPIN classifier

A prediction model was developed using a random forest algorithm to distinguish between benign, dominant, and recessive nsSNVs.

#### Training datasets

The benign training data used for the random forest algorithm are nsSNVs that are curated as benign in ClinVar and also annotated with either of the following: (i) criteria provided, multiple submitters, no conflicts or (ii) reviewed by expert panel or (iii) practice guideline (43). The pathogenic training dataset used is ExoVar, which is composed of Mendelian disease-causing mutations known to alter protein function curated from UniProt (17). These disease-causing mutations are subdivided into dominantly- and recessively-acting mutations using dominant and recessive disease-causing gene lists. The gene lists are derived from several publications based on curated information from OMIM (35–37). To focus on loss-of-function effects, a subset of genes that cause disease through haploinsufficiency are extracted from the dominant gene list. The haploinsufficient gene list is based on predictions generated using a model trained on genomic, evolutionary, functional, and network features (44).

#### Classification into three groups

Non-numeric, descriptive features are transformed into binary values (−1, 1) converting all the features into quantitative values and missing values for features are replaced with weighted averages of variants from all three classes (dominant, recessive and benign). The prediction model was built using a random forest algorithm with 10-fold cross-validation. For the haploinsufficient training data, one variant per gene was used for the benign and recessive datasets to reduce bias that may arise from overtraining on genes that contain numerous mutations. However, to create a more balanced training dataset, two variants per gene were used from the dominant class as shown in Table 1. For the ‘all dominant’ training data, one variant was used per gene for all three datasets (dominant, recessive, benign) as shown in Table 1. The variants were picked randomly and only the longest transcript containing the variant was used for the predictions.

**Table 1.** Number of variants used for training

Training set	# of genes	# of variants	# of training variants
Benign	292	1024	292
Haploinsufficient	119	598	238
Recessive	409	2413	409
‘All Dominant’	258	1298	258

This was repeated 40 times and multiclass AUC and precision values were calculated. Precision or positive predictive value is calculated as shown below:

\begin{eqnarray*} &&\frac{{{\rm True\ positives}}}{{{\rm True\ positives + False\ Positives}}}\ \end{eqnarray*}

Recall or sensitivity is calculated as:

\begin{eqnarray*} &&\frac{{{\rm True\ positives}}}{{{\rm True\ positives + False\ Negatives}}}\ \end{eqnarray*}

Importance plots are also generated to determine the contribution of each feature to accuracy of the out-of-bag (OOB) samples of the training data predictions by randomly permuting the feature and determining the decrease in mean accuracy (Supplementary Figure S1). In the randomForest R package, the mean decrease in accuracy is calculated as follows. First, OOB prediction error is calculated for each tree. Next, OOB error is calculated after permuting each variable (feature). Finally, to derive the mean decrease in accuracy, the difference between the two is averaged across all trees and normalized by the standard deviation of the differences.

### MAPPIN validation

Prediction scores for CADD (14) were obtained from http://cadd.gs.washington.edu/ and Eigen scores (45) were obtained from dbNSFP v3.0 (46). Variants for validation were obtained from publications from the Centers for Mendelian Genomics (CMG) (19) and the Deciphering Developmental Disorders Study (DDDS) (47). Pathogenic mutations tagged as ‘DM’ were obtained from HGMD (7).

### Usage

We provide a file that includes pre-computed MAPPIN scores for every single coding base that leads to a nonsynonymous change based on the GENCODE 19 gene annotation models and hg19 human reference genome. The scores for all three classes add up to one and each individual score represents the probability for the variant falling into that class. Additionally, a hg38 version of the MAPPIN predictions is available which was generated by lifting over the coordinates from hg19 to hg38. We also provide the standalone software for users who wish to modify or customize the method. MAPPIN has an easy-to-use command line interface allowing users to run it locally. Details for installing and executing MAPPIN software are provided under the data availability statement.

## RESULTS

### Feature annotations

For developing MAPPIN, we first annotated variants from curated data files with features that have been shown to be important for functional variant prediction. Evolutionary features and conservation metrics have repeatedly been demonstrated to be critical for distinguishing pathogenic from benign variants (9,10). These features have also been widely used in existing algorithms such as SIFT and PolyPhen. In addition to the commonly assessed conservation features such as GERP scores and dN/dS mutation rates, we also included gene-level metrics such as the presence of segmental duplications, pseudogenes, and importantly paralogs, which may be able to compensate for a gene’s function (48). Next, functional features that aid in variant interpretation such as protein domains, post-translational modifications, and expression in individual tissues from the Genotype Tissue Expression (GTEx) database (39) were annotated. Additionally, a value for overall tissue specificity was included using the Shannon entropy method (40,41). A third category of features included protein–protein interaction data, metabolic, genetic, and signaling networks (38). The principle of ‘guilt by association’ has been shown to be influenced by disease-inheritance modes and is highly relevant for recessive disease-causing genes (49). Finally, we annotated variants with allele frequency information from multiple populations using publicly available resources such as the 1000 Genomes Project (26), ESP6500 (27) and the Exome Aggregation Consortium (ExAC) (28). We also included a constraint metric, pLI, from ExAC, which denotes the probability that a gene is intolerant to loss-of-function variation (29), and thus is a measure of constraint and haploinsufficiency for a gene. A detailed description of all the features is included in the methods and a list of the features can be found in Supplementary Table S1.

### Training data

After assembling the features, we compiled training datasets of nsSNVs to develop a prediction model to distinguish between dominant, recessive, and benign variants. For pathogenic nsSNVs, we used the ExoVar training dataset (17), composed of 5,340 variants from UniProt that are associated with Mendelian diseases. The ExoVar pathogenic variants were further subdivided into 1,298 dominant and 2,413 recessive variants using curated lists of known dominant and recessive disease-causing genes (35–37). nsSNVs can affect function both through gain-of-function and loss-of-function mechanisms. However, it is more difficult to differentiate loss-of-function from gain-of-function events bioinformatically. Therefore, we created two versions of training datasets: a haploinsufficiency set focused on 598 nsSNVs that are believed to lead to dominantly inherited disease through molecular loss-of-function effects and an ‘all dominant’ set that includes all 1,298 variants in dominant disease-causing genes (44). The benign and recessive training data remained the same for the haploinsufficient and ‘all dominant’ prediction models. For benign variants we curated a list of 1,024 high-quality variants that are classified as benign in ClinVar (43) with stringent review and assertion criteria for clinical significance (2–4 gold stars).

### Random Forest classifier performance

**Figure 2.**
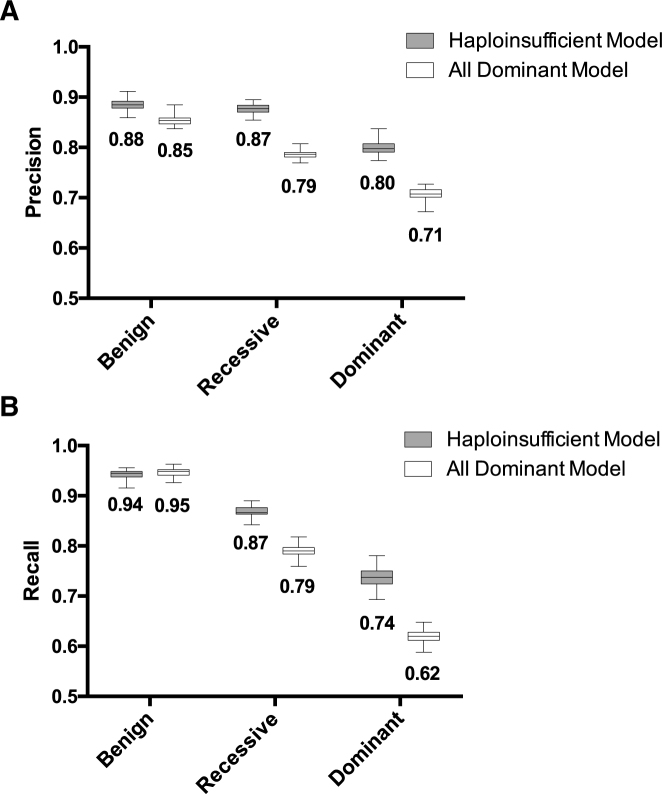
Precision (positive predictive value) and recall (sensitivity) values for the training datasets under the haploinsufficient and ‘all dominant’ models. Box plots with minimum and maximum of the precision (**A**) and recall (**B**) values are shown for both the haploinsufficient (gray) and ‘all dominant’ (white) models. Mean values for precision or recall are shown under each plot.

Next, we built a prediction model that not only distinguishes between pathogenic and benign variants, but also differentiates between heterozygous and homozygous or compound heterozygous disease-causing variants. A random forest machine learning method was used to classify the variants in the training datasets using 99 features spanning multiple categories. For the haploinsufficiency model, MAPPIN is able to classify the training variants into three classes (benign, dominant and recessive) with high accuracy. The average multiclass AUC with 10-fold cross-validation is 0.96. Precision or positive predictive values are 0.80, 0.87, and 0.88 for the dominant, recessive, and benign classes, respectively (Figure 2A). Additionally, the recall (sensitivity) values are 0.74, 0.87, and 0.94 for the dominant, recessive, and benign classes (Figure 2B). These metrics were also determined for the ‘all dominant’ model, which includes dominant disease-causing genes that may lead to a phenotype through haploinsufficiency, gain-of-function, or dominant-negative mechanisms. The multiclass AUC with 10-fold cross-validation is 0.91 and the precision values are 0.71, 0.79, and 0.85 for dominant, recessive, and benign classes, respectively (Figure 2A). The decrease in precision for the dominant group is reflected in the change in recall values for each class, which are 0.62 for dominant, 0.79 for recessive, and 0.95 for benign (Figure 2B). These results indicate a higher number of false positives and negatives with the ‘all dominant’ model presumably because MAPPIN is better at classifying nsSNVs with loss-of-function effects and performs better when trained with a restricted set of variants in haploinsufficient genes for the dominant class. Therefore, for further analysis and validation of the method we focused on the haploinsufficiency model.

Finally, we investigated whether subsets of features were sufficient for the predictions or if all features were required for the most accurate classifications. We evaluated the performance of the classifier using subsets of features for the haploinsufficient model as shown in Supplementary Table S2. No specific group of features (evolutionary, functional, network, etc.) is able to classify variants in all three classes well. Interestingly, allele frequency with pLI and the variant-specific features perform well in providing high confidence benign and recessive class predictions, but have very low precision values (0.43–0.64) for the dominant class (Supplementary Table S2). Combining all the features leads to a substantial improvement of prediction accuracy for dominant disease-causing variants.

### MAPPIN validation on pathogenic variants

#### Predicting pathogenicity and mode of inheritance of Mendelian variants

To validate MAPPIN, we first applied it to variants identified from the CMG studies (19), where mode of inheritance annotations are provided. The CMG set is composed of 68 disease-causing variants, which do not overlap with the training data. MAPPIN correctly predicts all 68 variants to be pathogenic, outperforming CADD and Eigen (Table 2 and Supplementary Table S4). The pathogenicity predictions do not change significantly even when MAPPIN is retrained by excluding disease-causing genes that are part of the CMG dataset from the training dataset (Table 2). Moreover, MAPPIN is able to accurately predict mode of inheritance for 45 of 64 variants with dominant or recessive annotations (Table 2). An interesting example from the CMG dataset is for a *de novo* variant in *SMAD2*, which is associated with an autosomal dominant form of congenital heart disease (Supplementary Table S3). For the variant in *SMAD2*, the dominant class score is 0.914, a very high score resulting in a confident dominant call. Other noteworthy predictions are for variants in *WDR62*, which result in microcephaly with or without cortical malformations in an autosomal recessive manner (19). For the mutations in *WDR62*, the recessive scores are 0.75 and 0.79 leading to high confidence classifications (Supplementary Table S3).

**Table 2.** MAPPIN prediction accuracy for two Mendelian datasets

Dataset	MAPPIN prediction accuracy	
	Pathogenicity	Inheritance
CMG	68/68 (100%)	45/64 (70.3%)
DDDS	138/158 (87.3%)	124/158 (78.5%)
CMG (genes not in training)	68/68 (100%)	45/64 (70.3%)
DDDS (genes not in training)	138/158 (87.3%)	125/158 (79.1%)

Table showing the prediction accuracies for pathogenicity and mode of inheritance for Mendelian validation datasets from the Centers for Mendelian Genomics (CMG) and the Deciphering Developmental Disorders Study (DDDS). CMG (genes not in training) and DDDS (genes not in training) are pathogenicity and mode of inheritance results after excluding all CMG and DDDS genes from the training data.

#### Classifying variants from the Deciphering Developmental Disorders Study

For a second validation set, we applied MAPPIN to variants identified in the DDDS (47). This study included 1133 children presenting with severe, undiagnosed developmental disorders and identified potential pathogenic mutations in 28% of children. Of the 158 autosomal variants that do not overlap with the training variants, 138 (87.3%) are predicted to be pathogenic by MAPPIN (Table 2 and Supplementary Table S4). Additionally, MAPPIN predicts 78.5% (124/158) accurately for mode of inheritance. Both the pathogenicity and mode of inheritance predictions did not change significantly when genes in DDDS were removed from training data and annotations (Table 2). Within the DDDS data, heterozygous disease-causing variants in *SCN2A*, which is associated with global developmental delay with seizures, are correctly identified as dominantly-acting with scores of 0.85 on average (Supplementary Table S3). Additionally, MAPPIN classifies variants within *TYR* as recessive with an average recessive score of 0.78 (Supplementary Table S3). Mutations in *TYR* in compound heterozygosity result in albinism, developmental stagnation, and autism (47).

#### Discrimination between dominant and recessive variants: MAPPIN versus other algorithms

**Figure 3.**
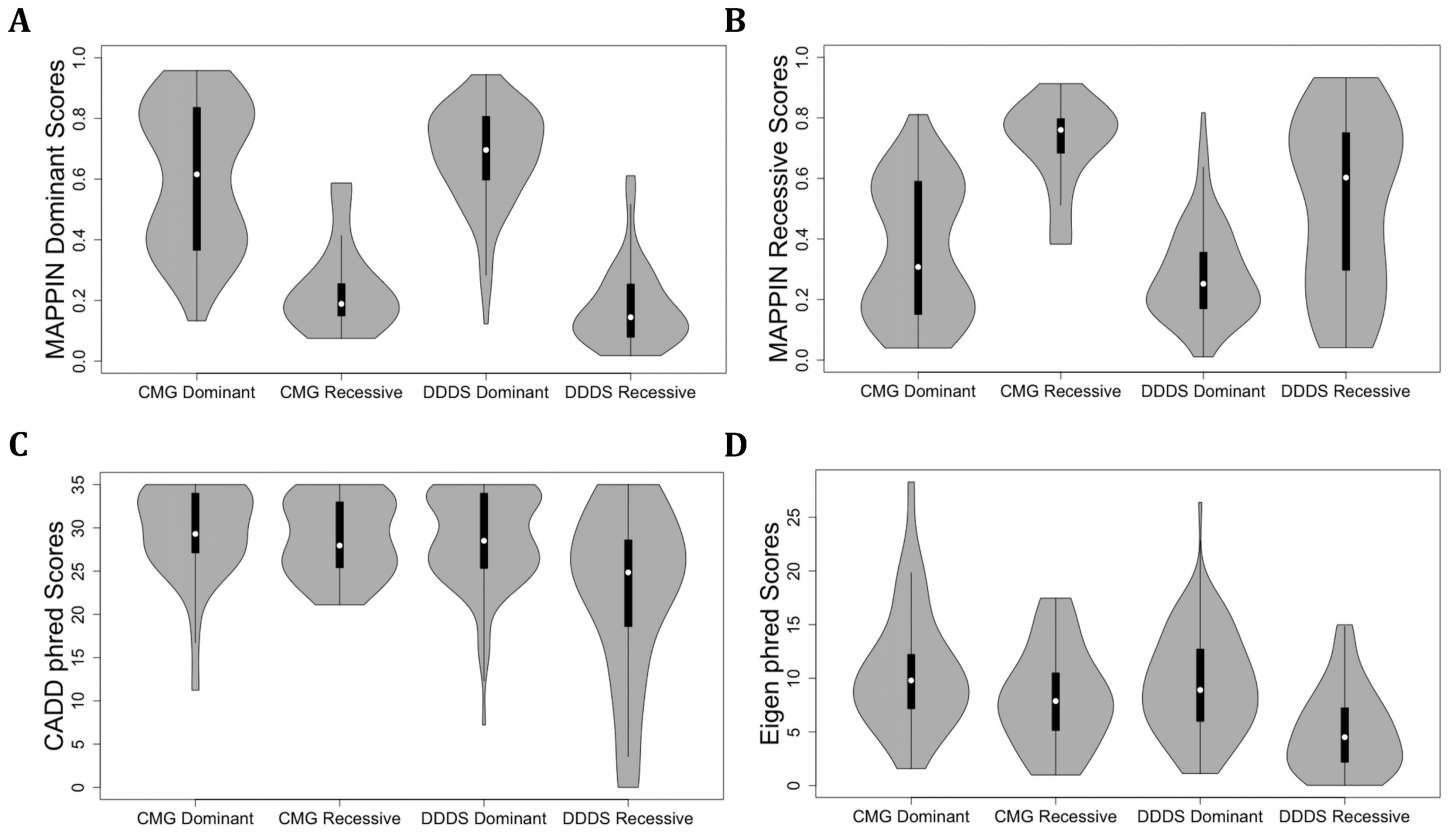
Violin plots of score distributions for CMG and DDDS dominant and recessive disease-causing variants. MAPPIN dominant (**A**) and recessive (**B**) class scores for CMG and DDDS genes annotated as dominant or recessive. CADD (**C**) and Eigen (**D**) phred scores for CMG and DDDS genes annotated as dominant or recessive.

Next, we analyzed dominant and recessive score distributions for the CMG and DDDS mutations to determine if MAPPIN scores discriminate between the two classes. Dominant class scores show a clear, significant difference between dominant and recessive mutations for both the CMG (*p* = 1.49e-08, Wilcoxon rank sum) and DDDS (*p* = 3.74e-23, Wilcoxon rank sum) datasets (Figure 3A). The mean dominant class score for dominant genes is 0.61 for CMG and 0.68 for DDDS, while the dominant class scores for the recessive genes are much lower with means of 0.23 and 0.18 for CMG and DDDS respectively. Additionally, MAPPIN recessive class scores also show a significant difference between dominant and recessive variants for both CMG (*p* = 2.48e-08) and DDDS datasets (*p* = 1.53e-06) (Figure 3B). Next, we assessed whether other newer or widely-used prediction algorithms not tested by Li *et al.* (17) could discriminate between dominant and recessive disease-causing variants. CADD C-scores (14) show no difference between dominant and recessive gene variants from CMG (*p* = 0.24, Wilcoxon rank sum) (Figure 3C). In contrast, CADD scores show a significant difference between dominant and recessive variants in the DDDS dataset (*p* = 4.19e-05, Wilcoxon rank sum). However, MAPPIN outperforms CADD with a p-value several orders of magnitude lower. Another recently published metric is the Eigen score (45). Similarly to CADD, the Eigen phred score obtained from dbNSFP (46) shows no discrimination between dominant and recessive variants from CMG (*p* = 0.11, Wilcoxon rank sum) (Figure 3D). The Eigen score is able to significantly differentiate between dominant and recessive variants from DDDS (*p* = 2.32e-08, Wilcoxon rank sum). Despite the statistically significant difference, CADD and Eigen score distributions for dominant and recessive variants overlap substantially (Figure 3C and D). It is evident that there is no clear cutoff to distinguish dominant from recessive variants on ranking the CMG and DDDS pathogenicity scores from highest to lowest for CADD and Eigen (Supplementary Table S5). This confirms that unlike CADD and Eigen, MAPPIN can distinguish between dominant and recessive Mendelian disease-causing variants in the CMG and DDDS datasets. However, it should be noted that unlike MAPPIN, CADD and Eigen were not trained using a multi-class classification to differentiate between dominant and recessive pathogenic mutations.

### Pathogenicity and mode of inheritance classification for HGMD variants

**Figure 4.**
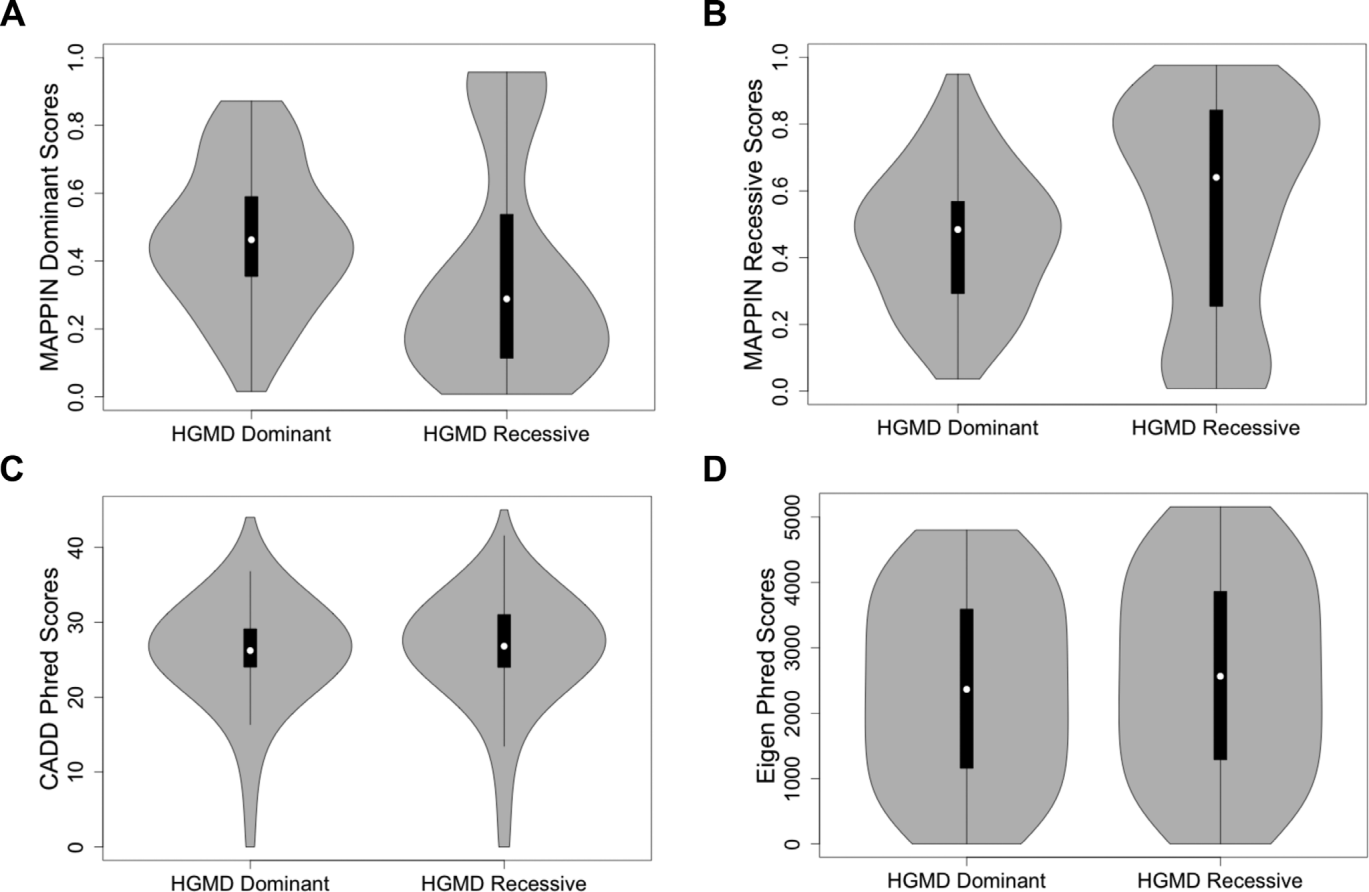
Violin plots of score distributions for HGMD variants in dominant and recessive genes. Training variants are excluded in the comparison and variants were subset using genes from Berg *et al.* MAPPIN dominant (**A**) and recessive (**B**) class scores for HGMD variants in dominant and recessive genes. CADD (**C**) and Eigen (**D**) phred scores for HGMD variants in dominant and recessive genes.

To ensure that discrimination by MAPPIN was not restricted to smaller Mendelian datasets, we utilized variants from HGMD as another validation dataset (7). To assign mode of inheritance to the HGMD variants, we classified the HGMD genes as dominant or recessive disease-causing using an orthogonal set of genes with known mode of inheritance from Berg *et al.* (50), which did not overlap with the genes in the training set. Furthermore, any HGMD mutations that overlapped with the training datasets from ExoVar (pathogenic) and ClinVar (benign) were excluded from this analysis. This unique set includes 5,395 variants from 164 dominant genes and 5,699 variants from 161 recessive genes. MAPPIN predicted 97.8% of the disease-causing mutations as pathogenic and this did not change significantly for cancer predisposition genes compared to all other genes (Supplementary Table S6). As would be expected, MAPPIN dominant class scores are significantly higher (*p* = 2.20e-206) for HGMD mutations falling into dominant genes versus those in recessive genes (Figure 4A). Complementary to this, MAPPIN recessive class scores are significantly higher (*p* = 3.55e-92) for mutations in recessive genes compared to dominant HGMD mutations (Figure 4B). Next, we evaluated this score discrimination with CADD and Eigen. CADD scores show a significant (*p* = 2.95e-07, Wilcoxon rank sum), but small difference between dominant and recessive variant mean scores (Figure 4C). However, this was in the opposite direction to the expectation that, generally, recessive mutations should have lower mean scores compared to dominant mutations. Dominant-acting variants are under higher selection constraint and therefore should have higher pathogenicity scores for both haploinsufficient loss-of-function mutations and gain-of-function mutations compared to recessive variants, where mutations on both alleles are required to result in a phenotype. Eigen scores show some difference (*p* = 2.32e-08, Wilcoxon rank sum) between dominant and recessive variants, but again this is contrary to expected results as recessive mutations have higher pathogenicity scores (Figure 4D). These data further demonstrate that MAPPIN is predicting pathogenicity with high confidence and unlike other prediction algorithms, it is able to discriminate between heterozygous and homozygous or compound heterozygous disease-causing mutations across multiple datasets.

## DISCUSSION

In this paper, we develop MAPPIN, a prediction algorithm that differentiates between benign, dominantly-acting, and recessively-acting nsSNVs. MAPPIN uses 99 annotated features to generate a prediction model based on a random forest classifier to provide pathogenicity and mode of inheritance predictions for nsSNVs. Combining several categories of features improves the prediction accuracy of the model and allows for substantially improved precision values for dominant disease-causing variants (Supplementary Table S2). The most important features consistently contributing to class assignment include evolutionary constraint metrics such as population allele frequencies, dN/dS rates, GERP scores, and the ExAC constraint score (pLI) (Supplementary Figure S1). This fits with previous literature and existing hypotheses that disease-causing genes should be under greater selection and that constraint may correlate with disease severity (18).

MAPPIN performs well with a multi-class AUC of 0.96 with 10-fold cross-validation and precision values of 0.80, 0.87, 0.88 for dominant, recessive, and benign classes, respectively (Figure 2A). MAPPIN is able to identify pathogenic variants accurately. More importantly, MAPPIN predicts mode of inheritance with >70% accuracy for disease-causing variants from the CMG and DDDS Mendelian discovery projects (Table 2). Moreover, MAPPIN scores show significant discrimination between heterozygous and homozygous or compound heterozygous disease-causing variants from both the Mendelian discovery projects and a larger collection of variants from HGMD.

In the validation data presented above, MAPPIN was run under the haploinsufficiency dataset model, which focuses on nsSNVs resulting in loss-of-function of the gene. As mentioned earlier, MAPPIN performs slightly better at modeling loss-of-function effects, although the precision and recall values (Figure 2A and B) under the ‘all dominant’ dataset model are still better than existing prediction algorithms (17). This inability to provide accurate predictions for gain-of-function or dominant-negative mutations is generally true of other prediction programs. For example, SIFT and PolyPhen were shown to be significantly better at predicting pathogenicity for loss-of-function nsSNVs compared to gain-of-function mutations (51). Flanagan *et al.* suggested that this may be because gain-of-function mutations have subtler effects on protein structure and are rare with a lower likelihood of being present in training data used by prediction algorithms. Additionally, this could occur because features to distinguish gain-of-function from loss-of-function nsSNVs are limited or unknown and are specific to the biological function of the gene making them difficult to model computationally.

A potential issue with nsSNV prediction programs is that they may suffer from two types of circularity: validation variant sets overlapping with training data (type 1) and/or a majority of genes in validation intersecting with the training model (type 2) (52). Variants in the CMG and DDDS validation data overlapping with those in training sets were removed before analyzing prediction accuracy for MAPPIN. Additionally, more than 75% of genes within the CMG and DDDS datasets are unique and not found in the training model. Finally, when overlapping CMG/DDDS genes are removed from the training dataset they do not affect the prediction accuracy significantly (Table 2). A similar approach was used with the HGMD data, where any variants overlapping with the training data or any genes present in the prediction model annotations were removed from the analysis.

In conclusion, the major advantage of MAPPIN over existing nsSNV prediction programs is its ability to predict mode of inheritance in addition to classifying variants as benign or pathogenic. Using MAPPIN, variants can be prioritized not only by pathogenicity, but by taking into account the underlying hypothesis for the inheritance pattern for the phenotype of interest (53). This is especially important as not taking genotype into account can result in incorrect classification of carrier heterozygous variants, which are only disease-causing when two copies are present, as equally damaging as dominant disease-causing mutations. We envision MAPPIN being used for a variety of analyses including family-based and population genetics approaches. In family-based analyses, the mode of inheritance predictions will aid in variant prioritization after filtering and segregation analysis in cases where there is not enough information or there are not enough family members to determine the inheritance pattern. In this situation, multiple inheritance models are tested (19,20) and MAPPIN could aid in variant prioritization through its ability to differentiate dominantly- and recessively-acting variants. Additionally, MAPPIN can be applied to dominant disease cases where discrete filtering is not sufficient to identify the causal variant as an individual can harbor thousands of heterozygous nsSNVs (22) and it is critical to differentiate the dominant variants from the carrier variants. For population genetics and association analyses, variant annotations and deleteriousness predictions by MAPPIN can be incorporated to support variant interpretation as well as understand phenotypic associations driven by heterozygotes versus homozygotes. Moreover, MAPPIN can aid in choosing variants for aggregation in gene burden based association analysis based on pathogenicity and mode of inheritance. Finally, the mode of inheritance prediction is also important for experimental design for *in vitro* or *in vivo* mouse modeling of the variants. This is essential because functional modeling is still necessary for confirmation of variant pathogenicity and determination of the mechanism of action.

## AVAILABILITY STATEMENT

The software, associated documentation and the associated data files required to run MAPPIN are available at https://doi.org/10.6084/m9.figshare.4639789. MAPPIN requires Linux (64-bit) or OS X 10.6 (64-bit) and above, Python 2.7+ or 3.x, and Perl. MAPPIN is provided under the CC BY-NC 4.0 license and is free to use and modify for non-commercial purposes. The pre-computed MAPPIN prediction score files for every single coding nonsynonymous change for the hg19 and hg38 genome builds are available at https://doi.org/10.6084/m9.figshare.4639789.

## Supplementary Material

Supplementary Data
