# Supplementary material for: MAPPIN: a method for annotating, predicting pathogenicity and mode of inheritance for nonsynonymous variants
Source: Nucleic Acids Res. 2017 Aug 25;45(18):10393–402. doi: 10.1093/nar/gkx730 (PMC5737764; doi:10.1093/nar/gkx730)
Supplement: Supplementary Data [file gkx730_supp.zip › nar-00689-z-2017-File007.pdf]

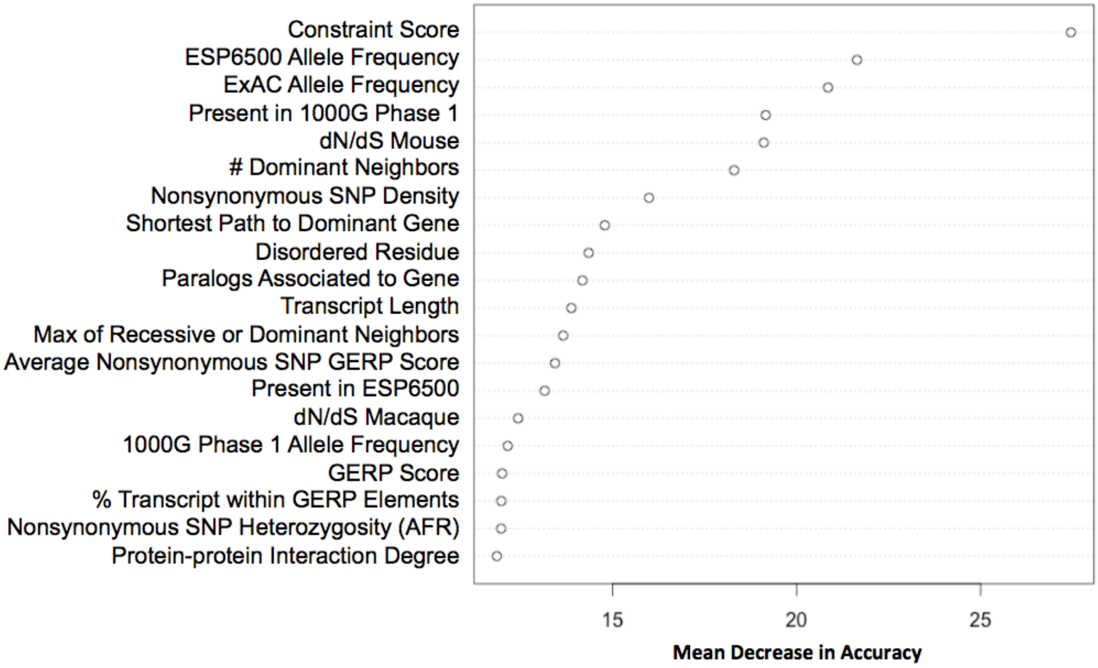

Supplementary Figure 1. Feature importance plot. The features were randomly permuted and the mean decrease in accuracy was measured for out-of-bag samples of the training data.

Number	Feature	Type	Feature Category
1	Chromosome	Details	-
2	Position	Details	-
3	rsID	Details	-
4	Reference allele	Details	-
5	Alternate allele	Details	-
6	Variant Score	Details	-
7	Pass	Details	-
8	Variant Details	Details	-
9	Gene	Details	-
10	Ensembl gene ID	Details	-
11	Ensembl transcript ID	Details	-
12	Transcript length	Gene	Functional
13	Longest transcript?	Gene	Functional
14	Partial?	Variant	Functional
15	GERP score	Variant	Evolutionary
16	In GERP element	Variant	Evolutionary
17	Segmental duplications	Gene	Evolutionary
18	PF domain	Variant	Functional
19	SSF domain	Variant	Functional
20	SM domain	Variant	Functional
21	Transmembrane helix domain	Variant	Functional
22	Signal peptide domain	Variant	Functional
23	Acetylation	Variant	Functional
24	Di-methylation	Variant	Functional
25	Methylation	Variant	Functional
26	Mono-methylation	Variant	Functional
27	O-GlcNAc	Variant	Functional
28	Phosphorylation	Variant	Functional
29	Sumoylation	Variant	Functional
30	Tri-methylation	Variant	Functional
31	Ubiquitination	Variant	Functional
32	Present in 1000G Phase1	Variant	Allele Frequency
33	1000G Phase1 allele frequency	Variant	Allele Frequency
34	1000G Phase1 Asian allele frequency	Variant	Allele Frequency
35	1000G Phase1 African allele frequency	Variant	Allele Frequency
36	1000G Phase1 European allele frequency	Variant	Allele Frequency
37	Present in ESP6500	Variant	Allele Frequency
38	ESP6500 allele frequency	Variant	Allele Frequency
39	Pseudogenes associated to transcript	Gene	Evolutionary
40	Paralogs associated to gene	Gene	Evolutionary

41	dN/dS (macaque)	Gene	Evolutionary
42	dN/dS (mouse)	Gene	Evolutionary
43	Shortest path to recessive gene	Gene	Network
44	Number of direct recessive neighbors	Gene	Network
45	Shortest path to dominant gene	Gene	Network
46	Number of direct dominant neighbors	Gene	Network
47	Shortest path to either dominant or recessive gene	Gene	Network
48	Number of recessive and dominant neighbors	Gene	Network
49	Disordered residue	Gene	Functional
50	Nonsynonymous SNP density	Gene	Evolutionary
51	Synonymous SNP density	Gene	Evolutionary
52	Average nonsynonymous variant GERP score	Gene	Evolutionary
53	% of nonsynonymous SNPs in GERP elements	Gene	Evolutionary
54	Average synonymous variant GERP score	Gene	Evolutionary
55	% of synonymous SNPs in GERP elements	Gene	Evolutionary
56	miRNA sites	Variant	Functional
57	Homozygous nonsynonymous SNPs/transcript length	Gene	Evolutionary
58	Homozygous synonymous SNPs/transcript length	Gene	Evolutionary
59	Homozygous premature stop SNPs/transcript length	Gene	Evolutionary
60	Homozygous spliceoverlap SNPs/transcript length	Gene	Evolutionary
61	% transcript overlapping with GERP elements	Gene	Evolutionary
62	Sum of heterozygosity for all non-synonymous SNPs / transcript length	Gene	Evolutionary
63	Sum of heterozygosity in ASN for all non-synonymous SNPs / transcript length	Gene	Evolutionary
64	Sum of heterozygosity in AMR for all non-synonymous SNPs / transcript length	Gene	Evolutionary
65	Sum of heterozygosity in AFR for all non-synonymous SNPs / transcript length	Gene	Evolutionary
66	Sum of heterozygosity in EUR for all non-synonymous SNPs / transcript length	Gene	Evolutionary
67	Sum of heterozygosity for all synonymous SNPs / transcript length	Gene	Evolutionary
68	Sum of heterozygosity in ASN for all synonymous SNPs / transcript length	Gene	Evolutionary
69	Sum of heterozygosity in AMR for all synonymous SNPs / transcript length	Gene	Evolutionary

70	Sum of heterozygosity in AFR for all synonymous SNPs / transcript length	Gene	Evolutionary
71	Sum of heterozygosity in EUR for all synonymous SNPs / transcript length	Gene	Evolutionary
72	PPI degree centrality	Gene	Network
73	Metabolic degree centrality	Gene	Network
74	Genetic network degree centrality	Gene	Network
75	Phosphorylation network degree centrality	Gene	Network
76	ENCODE regulatory network degree centrality	Gene	Network
77	Signaling network degree centrality	Gene	Network
78	SUPERNET_DEGREE	Gene	Network
79	# of networks gene is in	Gene	Network
80	# of simultaneously possible interactions for a gene	Gene	Network
81	Adipose tissue expression	Gene	Functional
82	Adrenal gland expression	Gene	Functional
83	Blood expression	Gene	Functional
84	Blood vessel expression	Gene	Functional
85	Brain expression	Gene	Functional
86	Breast expression	Gene	Functional
87	Colon expression	Gene	Functional
88	Esophagus expression	Gene	Functional
89	Fallopian tube expression	Gene	Functional
90	Heart expression	Gene	Functional
91	Kidney expression	Gene	Functional
92	Liver expression	Gene	Functional
93	Lung expression	Gene	Functional
94	Muscle expression	Gene	Functional
95	Nerve expression	Gene	Functional
96	Ovary expression	Gene	Functional
97	Pancreas expression	Gene	Functional
98	Pituitary expression	Gene	Functional
99	Prostate expression	Gene	Functional
100	Skin expression	Gene	Functional
101	Stomach expression	Gene	Functional
102	Testis expression	Gene	Functional
103	Thyroid expression	Gene	Functional
104	Uterus expression	Gene	Functional
105	Vagina expression	Gene	Functional
106	Overall tissue specific expression measured by Shannon entropy	Gene	Functional
107	Present in ExAC	Variant	Allele Frequency

108	ExAC allele frequency	Variant	Allele Frequency
109	ExAC pLI score	Gene	Allele Frequency
110	Splice score prediction	Variant	Functional

Supplementary Table 1. List of features used for annotation and prediction.

Feature Category	Benign	Recessive	Dominant
Evolutionary	0.53	0.63	0.52
Functional	0.47	0.63	0.48
Network	0.34	0.58	0.43
Frequency + pLI	0.91	0.87	0.59
Gene-based	0.36	0.61	0.59
Variant-based	0.91	0.81	0.64
All Features	0.88	0.87	0.80

Supplementary Table 2. Mean precision values obtained for the three classes of variants when different sets of features are used for training the classifier. The variants in haploinsufficient genes were used as the training dataset for the dominant class.

CHR	POS	REF	ALT	GENE	GENE ID	TRANSCRIPT ID	BENIGN	DOMINANT	RECESSIVE	MAX SCORE	CMG Annotation
chr17	54912344	G	C	DGKE	ENSG00000153933.5	ENST00000284061.3	0.0427	0.08405	0.87325	Recessive	Compound Het
chr2	233348866	G	A	ECEL1	ENSG00000171551.7	ENST00000304546.1	0.04075	0.1766	0.78265	Recessive	Compound Het
chr2	233348866	G	T	ECEL1	ENSG00000171551.7	ENST00000304546.1	0.0261	0.21335	0.76055	Recessive	Compound Het
chr2	233349788	T	C	ECEL1	ENSG00000171551.7	ENST00000304546.1	0.02615	0.2135	0.76035	Recessive	Compound Het
chr2	233350774	C	T	ECEL1	ENSG00000171551.7	ENST00000304546.1	0.02845	0.21445	0.7571	Recessive	Compound Het
chr11	71940602	G	C	INPPL1	ENSG00000165458.9	ENST00000298229.2	0.04265	0.5705	0.38685	Dominant	Compound Het
chr3	195975116	C	T	PCYT1A	ENSG00000161217.7	ENST00000419333.1	0.0167	0.252	0.7313	Recessive	Compound Het
chr2	73115586	A	G	SPR	ENSG00000116096.5	ENST00000234454.5	0.0788	0.14605	0.77515	Recessive	Compound Het
chr12	402269	G	A	KDM5A	ENSG00000073614.7	ENST00000399788.2	0.13615	0.55065	0.3132	Dominant	De Novo
chr18	10671630	C	A	PIEZO2	ENSG00000154864.7	ENST00000580640.1	0.0365	0.36195	0.60155	Recessive	De Novo
chr18	10671726	C	T	PIEZO2	ENSG00000154864.7	ENST00000580640.1	0.03705	0.365	0.59795	Recessive	De Novo
chr18	10671727	G	A	PIEZO2	ENSG00000154864.7	ENST00000580640.1	0.03705	0.365	0.59795	Recessive	De Novo
chr18	10696255	G	A	PIEZO2	ENSG00000154864.7	ENST00000580640.1	0.04425	0.36215	0.5936	Recessive	De Novo
chr18	10696261	G	A	PIEZO2	ENSG00000154864.7	ENST00000580640.1	0.04485	0.3654	0.58975	Recessive	De Novo
chr18	10762975	A	G	PIEZO2	ENSG00000154864.7	ENST00000580640.1	0.04405	0.36535	0.5906	Recessive	De Novo
chr18	10789112	T	C	PIEZO2	ENSG00000154864.7	ENST00000580640.1	0.0445	0.36705	0.58845	Recessive	De Novo
chr20	9389733	A	G	PLCB4	ENSG00000101333.12	ENST00000334005.3	0.02335	0.47155	0.5051	Recessive	De Novo
chr18	45375021	C	G	SMAD2	ENSG00000175387.11	ENST00000262160.6	0.0127	0.9145	0.0728	Dominant	De Novo
chr1	218609461	C	T	TGFB2	ENSG00000092969.7	ENST00000366929.4	0.01455	0.774	0.21145	Dominant	De Novo
chr5	133707309	G	C	UBE2B	ENSG00000119048.3	ENST00000265339.2	0.0076	0.75105	0.24135	Dominant	De Novo
chr12	95927820	C	A	USP44	ENSG00000136014.7	ENST00000258499.3	0.05615	0.133	0.81085	Recessive	De Novo
chr9	137005018	A	C	WDR5	ENSG00000196363.5	ENST00000358625.3	0.0912	0.6012	0.3076	Dominant	De Novo
chr1	110091460	G	C	GNAI3	ENSG00000065135.7	ENST00000369851.4	0.01265	0.61575	0.3716	Dominant	Dominant
chr11	533874	A	G	HRAS	ENSG00000174775.12	ENST00000311189.7	0.0534	0.72565	0.22095	Dominant	Dominant
chr18	24056567	T	G	KCTD1	ENSG00000134504.8	ENST00000417602.1	0.01135	0.8388	0.14985	Dominant	Dominant
chr18	24056581	G	T	KCTD1	ENSG00000134504.8	ENST00000417602.1	0.01135	0.8388	0.14985	Dominant	Dominant
chr18	24056603	C	T	KCTD1	ENSG00000134504.8	ENST00000417602.1	0.0113	0.8379	0.1508	Dominant	Dominant
chr18	24081101	G	T	KCTD1	ENSG00000134504.8	ENST00000417602.1	0.01135	0.8383	0.15035	Dominant	Dominant
chr18	24081102	T	G	KCTD1	ENSG00000134504.8	ENST00000417602.1	0.01135	0.8383	0.15035	Dominant	Dominant
chr18	24081108	G	T	KCTD1	ENSG00000134504.8	ENST00000417602.1	0.01325	0.83625	0.1505	Dominant	Dominant
chr18	24081108	G	A	KCTD1	ENSG00000134504.8	ENST00000417602.1	0.01325	0.83625	0.1505	Dominant	Dominant
chr18	24081108	G	C	KCTD1	ENSG00000134504.8	ENST00000417602.1	0.01325	0.83625	0.1505	Dominant	Dominant
chr18	24081111	G	T	KCTD1	ENSG00000134504.8	ENST00000417602.1	0.01605	0.83215	0.1518	Dominant	Dominant
chr18	24081142	G	A	KCTD1	ENSG00000134504.8	ENST00000417602.1	0.0167	0.8348	0.1485	Dominant	Dominant
chr1	115256529	A	G	NRAS	ENSG00000213281.4	ENST00000369535.4	0.0024	0.9578	0.0398	Dominant	Dominant
chr18	10671568	A	G	PIEZO2	ENSG00000154864.7	ENST00000580640.1	0.03705	0.365	0.59795	Recessive	Dominant
chr18	10671630	C	G	PIEZO2	ENSG00000154864.7	ENST00000580640.1	0.0365	0.36195	0.60155	Recessive	Dominant
chr18	10689744	G	A	PIEZO2	ENSG00000154864.7	ENST00000580640.1	0.03705	0.3652	0.59775	Recessive	Dominant
chr20	9364980	A	C	PLCB4	ENSG00000101333.12	ENST00000334005.3	0.02225	0.4676	0.51015	Recessive	Dominant
chr20	9389727	G	A	PLCB4	ENSG00000101333.12	ENST00000334005.3	0.02335	0.47155	0.5051	Recessive	Dominant
chr20	9389813	A	C	PLCB4	ENSG00000101333.12	ENST00000334005.3	0.02225	0.46765	0.5101	Recessive	Dominant
chr10	53227579	G	A	PRKG1	ENSG00000185532.10	ENST00000373980.4	0.01625	0.8467	0.13705	Dominant	Dominant
chr1	218609452	C	T	TGFB2	ENSG00000092969.7	ENST00000366929.4	0.01455	0.774	0.21145	Dominant	Dominant
chr1	218610765	C	A	TGFB2	ENSG00000092969.7	ENST00000366929.4	0.00835	0.77265	0.219	Dominant	Dominant
chr18	19761477	C	T	GATA6	ENSG00000141448.4	ENST00000269216.3	0.01595	0.3702	0.61385	Recessive	Dominant
chr19	6364528	A	C	CLPP	ENSG00000125656.4	ENST00000245816.4	0.02925	0.3331	0.63765	Recessive	Recessive
chr19	6364535	G	C	CLPP	ENSG00000125656.4	ENST00000245816.4	0.02545	0.3354	0.63915	Recessive	Recessive
chr17	54925356	G	C	DGKE	ENSG00000153933.5	ENST00000284061.3	0.012	0.075	0.913	Recessive	Recessive
chr2	233349182	C	T	ECEL1	ENSG00000171551.7	ENST00000304546.1	0.02645	0.21545	0.7581	Recessive	Recessive
chr13	39261788	G	C	FREM2	ENSG00000150893.9	ENST00000280481.7	0.32085	0.1372	0.54195	Recessive	Recessive
chr11	71944143	C	T	INPPL1	ENSG00000165458.9	ENST00000298229.2	0.02545	0.5872	0.38735	Dominant	Recessive
chr11	71944515	C	T	INPPL1	ENSG00000165458.9	ENST00000298229.2	0.03275	0.58445	0.3828	Dominant	Recessive
chr16	75665624	C	T	KARS	ENSG00000065427.10	ENST00000319410.5	0.0124	0.1887	0.7989	Recessive	Recessive
chr16	75670401	A	G	KARS	ENSG00000065427.10	ENST00000319410.5	0.01765	0.133	0.84935	Recessive	Recessive
chr3	195968956	T	C	PCYT1A	ENSG00000161217.7	ENST00000419333.1	0.01675	0.25415	0.7291	Recessive	Recessive
chr3	195975117	G	A	PCYT1A	ENSG00000161217.7	ENST00000419333.1	0.01685	0.25575	0.7274	Recessive	Recessive
chr5	148442585	T	C	SH3TC2	ENSG00000169247.7	ENST00000515425.1	0.0543	0.15965	0.78605	Recessive	Recessive
chr16	2546357	G	T	TBC1D24	ENSG00000162065.7	ENST00000293970.5	0.0392	0.15915	0.80165	Recessive	Recessive
chr16	2547027	G	C	TBC1D24	ENSG00000162065.7	ENST00000293970.5	0.0369	0.1674	0.7957	Recessive	Recessive
chr4	41259013	A	C	UCHL1	ENSG00000154277.8	ENST00000512788.1	0.0286	0.43095	0.54045	Recessive	Recessive
chr14	97312481	G	A	VRK1	ENSG00000100749.3	ENST00000216639.3	0.0471	0.1372	0.8157	Recessive	Recessive
chr14	97321690	G	A	VRK1	ENSG00000100749.3	ENST00000216639.3	0.03525	0.15245	0.8123	Recessive	Recessive
chr19	36558317	G	C	WDR62	ENSG00000075702.12	ENST00000401500.2	0.0379	0.16675	0.79535	Recessive	Recessive
chr19	36575580	G	A	WDR62	ENSG00000075702.12	ENST00000401500.2	0.11735	0.12715	0.7555	Recessive	Recessive

CHR	POS	REF	ALT	GENE	GENE ID	TRANSCRIPT ID	BENIGN	DOMINANT	RECESSIVE	MAX SCORE	DDDS Annotation
chr1	7797375	C	T	CAMTA1	ENSG00000171735.14	ENST00000303635.7	0.0364	0.6086	0.355	Dominant	Heterozygous
chr1	27089742	G	A	ARID1A	ENSG00000117713.13	ENST00000324856.7	0.15405	0.57225	0.2737	Dominant	Heterozygous
chr2	16085972	G	A	MYCN	ENSG00000134323.10	ENST00000281043.3	0.01625	0.6228	0.36095	Dominant	Heterozygous
chr2	50733693	G	A	NRXN1	ENSG00000179915.16	ENST00000404971.1	0.0534	0.6831	0.2635	Dominant	Heterozygous
chr2	60773293	G	T	BCL11A	ENSG00000119866.16	ENST00000335712.6	0.0176	0.70455	0.27785	Dominant	Heterozygous
chr2	60773348	C	A	BCL11A	ENSG00000119866.16	ENST00000335712.6	0.0176	0.7046	0.2778	Dominant	Heterozygous
chr2	60773352	T	G	BCL11A	ENSG00000119866.16	ENST00000335712.6	0.0176	0.7046	0.2778	Dominant	Heterozygous
chr2	166165214	T	G	SCN2A	ENSG00000136531.9	ENST00000283256.6	0.01455	0.8522	0.13325	Dominant	Heterozygous
chr2	166210714	T	C	SCN2A	ENSG00000136531.9	ENST00000283256.6	0.01485	0.8518	0.13335	Dominant	Heterozygous
chr2	166243484	T	A	SCN2A	ENSG00000136531.9	ENST00000283256.6	0.0148	0.8515	0.1337	Dominant	Heterozygous
chr2	166245954	G	A	SCN2A	ENSG00000136531.9	ENST00000283256.6	0.02065	0.83395	0.1454	Dominant	Heterozygous
chr2	166848020	A	C	SCN1A	ENSG00000144285.11	ENST00000303395.4	0.06395	0.65765	0.2784	Dominant	Heterozygous
chr2	200173680	C	T	SATB2	ENSG00000119042.12	ENST00000260926.5	0.01785	0.6348	0.34735	Dominant	Heterozygous
chr2	200193603	C	T	SATB2	ENSG00000119042.12	ENST00000260926.5	0.01785	0.63765	0.3445	Dominant	Heterozygous
chr2	200193611	C	T	SATB2	ENSG00000119042.12	ENST00000260926.5	0.01785	0.63765	0.3445	Dominant	Heterozygous

chr2	200213431	C	A	SATB2	ENSG00000119042.12	ENST00000260926.5	0.0176	0.63985	0.34255	Dominant	Heterozygous
chr2	223086091	G	A	PAX3	ENSG00000135903.14	ENST00000392069.2	0.0285	0.64085	0.33065	Dominant	Heterozygous
chr2	240036937	G	A	HDAC4	ENSG00000068024.12	ENST00000345617.3	0.13995	0.6319	0.22815	Dominant	Heterozygous
chr2	241724480	G	A	KIF1A	ENSG00000130294.10	ENST00000498729.2	0.02595	0.7792	0.19485	Dominant	Heterozygous
chr3	4687356	A	G	ITPR1	ENSG00000150995.13	ENST00000302640.8	0.0191	0.7781	0.2028	Dominant	Heterozygous
chr3	4687362	C	T	ITPR1	ENSG00000150995.13	ENST00000302640.8	0.04435	0.71595	0.2397	Dominant	Heterozygous
chr3	4856205	G	A	ITPR1	ENSG00000150995.13	ENST00000302640.8	0.06045	0.6883	0.25125	Dominant	Heterozygous
chr3	71026825	G	A	FOXP1	ENSG00000114861.14	ENST00000491238.1	0.0284	0.55345	0.41815	Dominant	Heterozygous
chr3	119133903	G	A	ARHGAP31	ENSG00000031081.6	ENST00000264245.4	0.04455	0.4906	0.46485	Dominant	Heterozygous
chr3	176750853	T	C	TBL1XR1	ENSG00000177565.11	ENST00000430069.1	0.0063	0.8935	0.1002	Dominant	Heterozygous
chr3	176755900	C	A	TBL1XR1	ENSG00000177565.11	ENST00000430069.1	0.00635	0.891	0.10265	Dominant	Heterozygous
chr3	181430372	T	C	SOX2	ENSG00000181449.2	ENST00000325404.1	0.01395	0.78495	0.2011	Dominant	Heterozygous
chr5	14397221	C	A	TRIO	ENSG00000038382.13	ENST00000344204.4	0.02055	0.6851	0.29435	Dominant	Heterozygous
chr5	14397222	C	T	TRIO	ENSG00000038382.13	ENST00000344204.4	0.02055	0.6851	0.29435	Dominant	Heterozygous
chr5	37017185	G	C	NIPBL	ENSG00000164190.12	ENST00000282516.8	0.054	0.66235	0.28365	Dominant	Heterozygous
chr5	74722257	G	A	COL4A3BP	ENSG00000113163.11	ENST00000380494.5	0.0234	0.59355	0.38305	Dominant	Heterozygous
chr5	139494382	A	T	PURA	ENSG00000185129.4	ENST00000331327.3	0.0099	0.6562	0.3339	Dominant	Heterozygous
chr5	176720974	G	A	NSD1	ENSG00000165671.14	ENST00000439151.2	0.0329	0.8799	0.0872	Dominant	Heterozygous
chr6	33400583	G	A	SYNGAP1	ENSG00000197283.8	ENST00000418600.2	0.03685	0.71825	0.2449	Dominant	Heterozygous
chr6	42975003	G	A	PPP2R5D	ENSG00000112640.10	ENST00000485511.1	0.0086	0.8268	0.1646	Dominant	Heterozygous
chr6	42975013	C	G	PPP2R5D	ENSG00000112640.10	ENST00000485511.1	0.0087	0.8265	0.1648	Dominant	Heterozygous
chr7	140453133	T	A	BRAF	ENSG00000157764.8	ENST00000288602.6	0.01265	0.82355	0.1638	Dominant	Heterozygous
chr7	140476811	C	T	BRAF	ENSG00000157764.8	ENST00000288602.6	0.01275	0.82355	0.1637	Dominant	Heterozygous
chr7	148504770	C	T	EZH2	ENSG00000106462.6	ENST00000320356.2	0.03065	0.8029	0.16645	Dominant	Heterozygous
chr7	148523618	G	A	EZH2	ENSG00000106462.6	ENST00000320356.2	0.03025	0.80635	0.1634	Dominant	Heterozygous
chr8	61732609	G	A	CHD7	ENSG00000171316.7	ENST00000423902.2	0.03365	0.77725	0.1891	Dominant	Heterozygous
chr9	2058457	G	A	SMARCA2	ENSG00000080503.15	ENST00000349721.2	0.10065	0.69385	0.2055	Dominant	Heterozygous
chr9	2060867	C	T	SMARCA2	ENSG00000080503.15	ENST00000349721.2	0.1219	0.677	0.2011	Dominant	Heterozygous
chr9	2060868	G	A	SMARCA2	ENSG00000080503.15	ENST00000349721.2	0.1219	0.677	0.2011	Dominant	Heterozygous
chr9	2181575	G	A	SMARCA2	ENSG00000080503.15	ENST00000349721.2	0.0938	0.6987	0.2075	Dominant	Heterozygous
chr9	98241382	A	C	PTCH1	ENSG00000185920.11	ENST00000331920.6	0.0079	0.92895	0.06315	Dominant	Heterozygous
chr9	130428485	G	A	STXBP1	ENSG00000136854.13	ENST00000373302.3	0.01275	0.8066	0.18065	Dominant	Heterozygous
chr9	130444768	G	T	STXBP1	ENSG00000136854.13	ENST00000373302.3	0.01265	0.8064	0.18095	Dominant	Heterozygous
chr9	130982480	C	T	DNM1	ENSG00000106976.14	ENST00000372923.3	0.0219	0.73925	0.23885	Dominant	Heterozygous
chr9	130984491	A	T	DNM1	ENSG00000106976.14	ENST00000372923.3	0.01685	0.76105	0.2221	Dominant	Heterozygous
chr9	130985129	C	G	DNM1	ENSG00000106976.14	ENST00000372923.3	0.01935	0.76505	0.2156	Dominant	Heterozygous
chr9	131388821	C	G	SPTAN1	ENSG00000197694.9	ENST00000358161.5	0.0254	0.8433	0.1313	Dominant	Heterozygous
chr10	76789597	G	C	KAT6B	ENSG00000156650.8	ENST00000287239.4	0.0388	0.67875	0.28245	Dominant	Heterozygous
chr10	76789623	G	A	KAT6B	ENSG00000156650.8	ENST00000287239.4	0.0388	0.67875	0.28245	Dominant	Heterozygous
chr10	123260357	G	A	FGFR2	ENSG00000066468.16	ENST00000457416.2	0.02585	0.7212	0.25295	Dominant	Heterozygous
chr11	64428503	C	T	NRXN2	ENSG00000110076.14	ENST00000265459.6	0.0702	0.5822	0.3476	Dominant	Heterozygous
chr12	13720098	C	G	GRIN2B	ENSG00000273079.1	ENST00000609686.1	0.0132	0.73405	0.25275	Dominant	Heterozygous
chr12	49420264	A	C	KMT2D	ENSG00000167548.10	ENST00000301067.7	0.04115	0.75215	0.2067	Dominant	Heterozygous
chr12	49445799	G	A	KMT2D	ENSG00000167548.10	ENST00000301067.7	0.05275	0.736	0.21125	Dominant	Heterozygous
chr12	52082570	A	G	SCN8A	ENSG00000196876.9	ENST00000354534.6	0.02225	0.5491	0.42865	Dominant	Heterozygous
chr12	52200143	G	A	SCN8A	ENSG00000196876.9	ENST00000354534.6	0.02215	0.54825	0.4296	Dominant	Heterozygous
chr12	112888168	T	G	PTPN11	ENSG00000179295.11	ENST00000351677.2	0.0285	0.8041	0.1674	Dominant	Heterozygous
chr12	112926887	G	A	PTPN11	ENSG00000179295.11	ENST00000351677.2	0.0285	0.80195	0.16955	Dominant	Heterozygous
chr12	116408461	G	A	MED13L	ENSG00000123066.3	ENST00000281928.3	0.0292	0.61395	0.35685	Dominant	Heterozygous
chr12	116413012	C	T	MED13L	ENSG00000123066.3	ENST00000281928.3	0.0291	0.61555	0.35535	Dominant	Heterozygous
chr14	21871618	T	G	CHD8	ENSG00000100888.8	ENST00000399982.2	0.02945	0.75945	0.2111	Dominant	Heterozygous
chr14	36987163	G	C	NKX2-1	ENSG00000136352.13	ENST00000354822.5	0.0315	0.63045	0.33805	Dominant	Heterozygous
chr15	48738953	T	C	FBN1	ENSG00000166147.9	ENST00000316623.5	0.0492	0.8109	0.1399	Dominant	Heterozygous
chr15	93499821	C	T	CHD2	ENSG00000173575.14	ENST00000394196.4	0.0231	0.6305	0.3464	Dominant	Heterozygous
chr16	3779449	G	A	CREBBP	ENSG00000005339.8	ENST00000262367.5	0.2147	0.775	0.0103	Dominant	Heterozygous
chr16	3779563	G	C	CREBBP	ENSG00000005339.8	ENST00000262367.5	0.1668	0.8135	0.0197	Dominant	Heterozygous
chr16	30718647	C	G	SRCAP	ENSG00000080603.12	ENST00000262518.4	0.03595	0.6402	0.32385	Dominant	Heterozygous
chr16	56309901	T	G	GNAO1	ENSG00000087258.9	ENST00000262493.6	0.02755	0.5375	0.43495	Dominant	Heterozygous
chr16	56370674	C	T	GNAO1	ENSG00000087258.9	ENST00000262493.6	0.02755	0.53725	0.4352	Dominant	Heterozygous
chr16	67645953	A	C	CTCF	ENSG00000102974.10	ENST00000264010.4	0.0082	0.82015	0.17165	Dominant	Heterozygous
chr16	67654615	C	T	CTCF	ENSG00000102974.10	ENST00000264010.4	0.0083	0.8236	0.1681	Dominant	Heterozygous
chr16	67654646	C	T	CTCF	ENSG00000102974.10	ENST00000264010.4	0.0091	0.8209	0.17	Dominant	Heterozygous
chr17	29654736	C	T	NF1	ENSG00000196712.12	ENST00000358273.4	0.0123	0.94425	0.04345	Dominant	Heterozygous
chr18	42531866	C	G	SETBP1	ENSG00000152217.12	ENST00000282030.5	0.0288	0.50875	0.46245	Dominant	Heterozygous
chr18	48604676	A	G	SMAD4	ENSG00000141646.9	ENST00000342988.3	0.01085	0.9262	0.06295	Dominant	Heterozygous
chr19	11132405	G	A	SMARCA4	ENSG00000127616.13	ENST00000358026.2	0.0573	0.7743	0.1684	Dominant	Heterozygous
chr19	42474691	C	T	ATP1A3	ENSG00000105409.11	ENST00000441343.1	0.04565	0.53705	0.4173	Dominant	Heterozygous
chr19	42489099	C	T	ATP1A3	ENSG00000105409.11	ENST00000441343.1	0.04105	0.5455	0.41345	Dominant	Heterozygous
chr19	52715971	C	T	PPP2R1A	ENSG00000105568.13	ENST00000322088.6	0.0295	0.83725	0.13325	Dominant	Heterozygous
chr19	52715979	C	T	PPP2R1A	ENSG00000105568.13	ENST00000322088.6	0.0295	0.83725	0.13325	Dominant	Heterozygous
chr20	62069997	G	A	KCNQ2	ENSG00000075043.13	ENST00000354587.3	0.0515	0.51125	0.43725	Dominant	Heterozygous
chr20	62071037	C	T	KCNQ2	ENSG00000075043.13	ENST00000354587.3	0.0452	0.4948	0.46	Dominant	Heterozygous
chr20	62073782	C	T	KCNQ2	ENSG00000075043.13	ENST00000354587.3	0.0507	0.48145	0.46785	Dominant	Heterozygous
chr20	62073787	G	A	KCNQ2	ENSG00000075043.13	ENST00000354587.3	0.0507	0.48145	0.46785	Dominant	Heterozygous
chr21	38858872	T	C	DYRK1A	ENSG00000157540.15	ENST00000398960.2	0.01615	0.81325	0.1706	Dominant	Heterozygous
chr21	38862672	A	T	DYRK1A	ENSG00000157540.15	ENST00000398960.2	0.01645	0.81165	0.1719	Dominant	Heterozygous
chr21	38865403	T	C	DYRK1A	ENSG00000157540.15	ENST00000398960.2	0.0165	0.8131	0.1704	Dominant	Heterozygous
chr4	126240408	A	G	FAT4	ENSG00000196159.7	ENST00000394329.3	0.0509	0.61125	0.33785	Dominant	Compound Het
chr4	126373642	G	A	FAT4	ENSG00000196159.7	ENST00000394329.3	0.06375	0.52835	0.4079	Dominant	Compound Het
chr8	144990697	T	A	PLEC	ENSG00000178209.10	ENST00000322810.4	0.0513	0.5838	0.3649	Dominant	Compound Het
chr8	144996236	C	G	PLEC	ENSG00000178209.10	ENST00000322810.4	0.3248	0.33765	0.33755	Dominant	Compound Het

chr1	52863470	C	A	ORC1	ENSG00000085840.8	ENST00000371566.1	0.16815	0.0892	0.74265	Recessive	Homozygous
chr1	220364620	C	T	RAB3GAP2	ENSG00000118873.11	ENST00000358951.2	0.04135	0.27215	0.6865	Recessive	Compound Het
chr1	220375711	C	G	RAB3GAP2	ENSG00000118873.11	ENST00000358951.2	0.0994	0.26125	0.63935	Recessive	Compound Het
chr2	73747141	A	C	ALMS1	ENSG00000116127.13	ENST00000264448.6	0.11985	0.147	0.73315	Recessive	Compound Het
chr2	74058095	C	T	STAMBP	ENSG00000124356.11	ENST00000339566.3	0.10675	0.261	0.63225	Recessive	Compound Het
chr2	74071966	A	G	STAMBP	ENSG00000124356.11	ENST00000339566.3	0.0698	0.27715	0.65305	Recessive	Compound Het
chr2	220431609	C	G	OBSL1	ENSG00000124006.10	ENST00000404537.1	0.08155	0.3126	0.60585	Recessive	Compound Het
chr4	15602948	T	C	CC2D2A	ENSG00000048342.11	ENST00000413206.1	0.10275	0.2083	0.68895	Recessive	Homozygous
chr5	177034310	C	T	B4GALT7	ENSG00000027847.9	ENST00000029410.5	0.2289	0.17095	0.60015	Recessive	Compound Het
chr5	177035541	G	A	B4GALT7	ENSG00000027847.9	ENST00000029410.5	0.0674	0.1502	0.7824	Recessive	Compound Het
chr5	177035995	C	T	B4GALT7	ENSG00000027847.9	ENST00000029410.5	0.0693	0.15375	0.77695	Recessive	Compound Het
chr6	88239290	A	T	RARS2	ENSG00000146282.13	ENST00000369536.5	0.0134	0.0892	0.8974	Recessive	Compound Het
chr7	66098307	A	G	KCTD7	ENSG00000243335.4	ENST00000275532.3	0.075	0.30965	0.61535	Recessive	Compound Het
chr8	38111143	A	G	DDHD2	ENSG00000085788.9	ENST00000397166.2	0.03295	0.07085	0.8962	Recessive	Compound Het
chr8	38111197	A	G	DDHD2	ENSG00000085788.9	ENST00000397166.2	0.01945	0.0826	0.89795	Recessive	Compound Het
chr8	141321437	G	A	TRAPPC9	ENSG00000167632.10	ENST00000389328.4	0.05455	0.15795	0.7875	Recessive	Compound Het
chr8	141445217	G	A	TRAPPC9	ENSG00000167632.10	ENST00000389328.4	0.1401	0.14175	0.71815	Recessive	Compound Het
chr9	37783990	T	G	EXOSC3	ENSG00000107371.8	ENST00000327304.5	0.1472	0.1203	0.7325	Recessive	Homozygous
chr10	50691439	G	A	ERCC6	ENSG00000225830.6	ENST00000355832.5	0.0428	0.15555	0.80165	Recessive	Compound Het
chr10	89473067	G	C	PAPSS2	ENSG00000198682.8	ENST00000456849.1	0.00855	0.0653	0.92615	Recessive	Compound Het
chr10	89475544	G	A	PAPSS2	ENSG00000198682.8	ENST00000456849.1	0.25085	0.1584	0.59075	Recessive	Compound Het
chr11	71148919	T	C	DHCR7	ENSG00000172893.11	ENST00000355527.3	0.0377	0.18245	0.77985	Recessive	Compound Het
chr11	88911734	C	A	TYR	ENSG00000077498.8	ENST00000263321.5	0.2282	0.07605	0.69575	Recessive	Compound Het
chr11	88961072	C	A	TYR	ENSG00000077498.8	ENST00000263321.5	0.0871	0.05645	0.85645	Recessive	Compound Het
chr11	121008311	G	C	TECTA	ENSG00000109927.5	ENST00000264037.2	0.1061	0.4106	0.4833	Recessive	Compound Het
chr12	88500654	G	A	CEP290	ENSG00000198707.10	ENST00000309041.7	0.1417	0.29685	0.56145	Recessive	Compound Het
chr13	101756915	G	A	NALCN	ENSG00000102452.11	ENST00000251127.6	0.10085	0.2753	0.62385	Recessive	Compound Het
chr15	40707168	G	A	IVD	ENSG00000128928.4	ENST00000487418.2	0.02415	0.25185	0.724	Recessive	Homozygous
chr16	56545126	C	T	BBS2	ENSG00000125124.7	ENST00000245157.5	0.01125	0.0557	0.93305	Recessive	Compound Het
chr18	43447589	A	G	EPG5	ENSG00000152223.8	ENST00000282041.5	0.04035	0.17255	0.7871	Recessive	Homozygous
chr18	67684696	A	G	RTTN	ENSG00000176225.8	ENST00000255674.6	0.0246	0.0678	0.9076	Recessive	Compound Het
chr19	7592776	G	A	MCOLN1	ENSG00000090674.11	ENST00000264079.6	0.12115	0.0601	0.81875	Recessive	Compound Het
chr17	36895854	G	A	PCGF2	ENSG00000056661.9	ENST00000360797.2	0.0433	0.37165	0.58505	Recessive	Heterozygous
chr17	78172347	C	T	CARD14	ENSG00000141527.12	ENST00000344227.2	0.06035	0.1225	0.81715	Recessive	Heterozygous
chr15	26806242	A	G	GABRB3	ENSG00000166206.9	ENST00000541819.2	0.02115	0.32385	0.655	Recessive	Heterozygous
chr12	22061091	C	T	ABCC9	ENSG00000069431.6	ENST00000261200.4	0.03265	0.3805	0.58685	Recessive	Heterozygous
chr11	65978677	C	T	PACS1	ENSG00000175115.7	ENST00000320580.4	0.07015	0.45215	0.4777	Recessive	Heterozygous
chr6	157454286	G	T	ARID1B	ENSG00000049618.17	ENST00000367148.1	0.0324	0.4811	0.4865	Recessive	Heterozygous
chr2	121708956	G	A	GLI2	ENSG00000074047.16	ENST00000361492.4	0.1674	0.2909	0.5417	Recessive	Heterozygous
chr1	103427786	A	T	COL11A1	ENSG00000060718.14	ENST00000358392.2	0.0846	0.23905	0.67635	Recessive	Heterozygous
chr1	147380372	T	G	GJA8	ENSG00000121634.4	ENST00000240986.4	0.0389	0.2475	0.7136	Recessive	Heterozygous
chr20	62071032	G	C	KCNQ2	ENSG00000075043.13	ENST00000354587.3	0.0537	0.4724	0.4739	Recessive	Heterozygous
chr1	22200454	G	T	HSPG2	ENSG00000142798.12	ENST00000374695.3	0.55315	0.2306	0.21625	Tolerant	Compound Het
chr1	22205601	T	C	HSPG2	ENSG00000142798.12	ENST00000374695.3	0.90115	0.05695	0.0419	Tolerant	Compound Het
chr1	152284382	C	T	FLG	ENSG00000143631.10	ENST00000368799.1	0.7941	0.07275	0.13315	Tolerant	Compound Het
chr1	236966848	G	A	MTR	ENSG00000116984.8	ENST00000366577.5	0.941	0.01815	0.04085	Tolerant	Homozygous
chr2	73675690	A	G	ALMS1	ENSG00000116127.13	ENST00000264448.6	0.8139	0.0452	0.1409	Tolerant	Compound Het
chr2	170022537	C	T	LRP2	ENSG00000081479.8	ENST00000263816.3	0.713	0.1398	0.1472	Tolerant	Compound Het
chr2	170068598	C	T	LRP2	ENSG00000081479.8	ENST00000263816.3	0.4951	0.28105	0.22385	Tolerant	Compound Het
chr2	170088242	G	A	LRP2	ENSG00000081479.8	ENST00000263816.3	0.7063	0.1701	0.1236	Tolerant	Compound Het
chr2	170103472	G	A	LRP2	ENSG00000081479.8	ENST00000263816.3	0.78335	0.1287	0.08795	Tolerant	Compound Het
chr2	220432804	G	A	OBSL1	ENSG00000124006.10	ENST00000404537.1	0.8339	0.07925	0.08685	Tolerant	Compound Het
chr4	122765152	T	C	BBS7	ENSG00000138686.5	ENST00000264499.4	0.56805	0.1132	0.31875	Tolerant	Compound Het
chr7	121733190	G	A	AASS	ENSG00000008311.10	ENST00000393376.1	0.75475	0.0771	0.16815	Tolerant	Homozygous
chr10	50678615	T	C	ERCC6	ENSG00000225830.6	ENST00000355832.5	0.52875	0.11735	0.3539	Tolerant	Compound Het
chr12	88519133	C	T	CEP290	ENSG00000198707.10	ENST00000309041.7	0.5927	0.0765	0.3308	Tolerant	Compound Het
chr14	89338702	A	G	TTC8	ENSG00000165533.14	ENST00000338104.6	0.537	0.17525	0.28775	Tolerant	Homozygous
chr15	49033814	G	C	CEP152	ENSG00000103995.9	ENST00000380950.2	0.461	0.1193	0.4197	Tolerant	Compound Het
chr15	49037112	G	C	CEP152	ENSG00000103995.9	ENST00000380950.2	0.5163	0.1113	0.3724	Tolerant	Compound Het
chr15	49060434	T	C	CEP152	ENSG00000103995.9	ENST00000380950.2	0.90815	0.0324	0.05945	Tolerant	Compound Het
chr16	88871972	G	A	CDT1	ENSG00000167513.4	ENST00000301019.4	0.7352	0.11545	0.14935	Tolerant	Compound Het
chr16	89825107	G	C	FANCA	ENSG00000187741.10	ENST00000389301.3	0.5932	0.1071	0.2997	Tolerant	Compound Het

Supplementary Table 3. MAPPIN prediction scores for all three classes for the variants in CMG and DDDS datasets.

Dataset	MAPPIN Prediction Accuracy	CADD (>=15) Prediction Accuracy	Eigen (>=5) Prediction Accuracy
CMG (Fig. 3)	68/68 (100%)	59/60 (98.3%)	50/60 (83.3%)
DDDS (Fig. 3)	138/158 (87.3%)	145/158 (91.8%)	109/158 (69.0%)
HGMD (Fig. 4)	56559/57850 (97.8%)	54241/57793 (93.9%)	34040/57993 (58.7%)

Supplementary Table 4. Pathogenicity prediction accuracies are shown for all three validation datasets: CMG, DDDS, HGMD. CADD and Eigen phred scores were obtained from dbNSFP.

CHR	POS	REF	ALT	CADD Score	CMG Annotation
1	218609452	C	T	35	Dominant
1	218609461	C	T	35	De Novo
10	53227579	G	A	35	Dominant
11	71944515	C	T	35	Recessive
18	19761477	C	T	35	Dominant
20	9389727	G	A	35	Dominant
12	402269	G	A	34	De Novo
14	97312481	G	A	34	Recessive
16	75665624	C	T	34	Recessive
18	10671630	C	A	34	De Novo
18	10671630	C	G	34	Dominant
18	10671726	C	T	34	De Novo
18	10671727	G	A	34	De Novo
18	24081111	G	T	34	Dominant
19	36575580	G	A	34	Recessive
4	41259013	A	C	34	Recessive
1	110091460	G	C	33	Dominant
14	97321690	G	A	33	Recessive
17	54925356	G	C	33	Recessive
18	24056603	C	T	33	Dominant
18	45375021	C	G	33	De Novo
11	71944143	C	T	32	Recessive
18	10689744	G	A	32	Dominant
18	24056581	G	T	32	Dominant
18	24081108	G	A	32	Dominant
19	36558317	G	C	32	Recessive
2	233348866	G	A	32	Compound Het
13	39261788	G	C	29.5	Recessive
18	24081142	G	A	29.3	Dominant
18	24081108	G	T	29	Dominant
20	9389733	A	G	28.5	De Novo
5	133707309	G	C	28.2	De Novo
18	24081108	G	C	28	Dominant
2	233350774	C	T	28	Compound Het
18	24081101	G	T	27.9	Dominant
2	233348866	G	T	27.9	Compound Het
16	75670401	A	G	27.8	Recessive
1	218610765	C	A	27.7	Dominant
18	24056567	T	G	27.3	Dominant
18	24081102	T	G	27.3	Dominant
18	10671568	A	G	26.9	Dominant
2	233349788	T	C	26.6	Compound Het
20	9364980	A	C	26.5	Dominant
19	6364535	G	C	26.4	Recessive
20	9389813	A	C	26.3	Dominant
16	2547027	G	C	26.1	Recessive
18	10762975	A	G	25.8	De Novo
19	6364528	A	C	25.4	Recessive
2	233349182	C	T	25.4	Recessive
18	10789112	T	C	24.5	De Novo
18	10696255	G	A	24.4	De Novo
18	2763729	T	C	24.3	Digenic
16	2546357	G	T	24.2	Recessive
2	73115586	A	G	24.2	Compound Het
18	10696261	G	A	23.4	De Novo
11	71940602	G	C	22.8	Compound Het
5	148442585	T	C	22.6	Recessive
17	54912344	G	C	21.1	Compound Het
12	95927820	C	A	17.84	De Novo
9	137005018	A	C	11.23	De Novo

CHR	POS	REF	ALT	Eigen Score	CMG Annotation
20	9389727	G	A	28.28369	Dominant
10	53227579	G	A	21.37921	Dominant
20	9364980	A	C	19.49376	Dominant
1	110091460	G	C	18.56284	Dominant
20	9389733	A	G	18.35861	De Novo
16	75665624	C	T	17.4636	Recessive
20	9389813	A	C	16.59783	Dominant
16	75670401	A	G	16.22202	Recessive
18	45375021	C	G	15.6074	De Novo
11	71944143	C	T	15.60146	Recessive
19	6364535	G	C	13.21607	Recessive
1	218610765	C	A	12.89652	Dominant
19	36558317	G	C	12.68342	Recessive
18	19761477	C	T	12.57637	Dominant
19	6364528	A	C	11.90388	Recessive
18	10689744	G	A	11.87636	Dominant
18	24081108	G	T	11.64754	Dominant
18	10671727	G	A	11.54912	De Novo
18	24056581	G	T	11.04159	Dominant
18	10671630	C	G	10.89124	Dominant
18	10671630	C	A	10.88377	De Novo
18	10671726	C	T	10.0668	De Novo
14	97321690	G	A	10.03812	Recessive
5	133707309	G	C	9.981146	De Novo
18	10762975	A	G	9.800605	De Novo
4	41259013	A	C	9.404865	Recessive
17	54925356	G	C	9.162892	Recessive
18	24056603	C	T	9.026028	Dominant
2	233349788	T	C	8.733541	Compound Het
12	402269	G	A	8.339682	De Novo
1	218609452	C	T	8.285764	Dominant
13	39261788	G	C	8.219731	Recessive
1	218609461	C	T	7.995812	De Novo
11	71944515	C	T	7.979619	Recessive
18	24081108	G	C	7.952439	Dominant
19	36575580	G	A	7.765035	Recessive
18	24081142	G	A	7.650493	Dominant
18	24081102	T	G	7.412012	Dominant
18	10671568	A	G	7.201119	Dominant
18	24056567	T	G	7.114009	Dominant
2	233348866	G	A	7.081037	Compound Het
18	24081108	G	A	7.046663	Dominant
16	2546357	G	T	7.000119	Recessive
18	24081101	G	T	6.717477	Dominant
14	97312481	G	A	6.633257	Recessive
2	233348866	G	T	6.315883	Compound Het
18	10696255	G	A	6.096745	De Novo
18	24081111	G	T	5.761204	Dominant
2	233350774	C	T	5.201472	Compound Het
18	10789112	T	C	5.118934	De Novo
18	10696261	G	A	4.908886	De Novo
16	2547027	G	C	4.90561	Recessive
2	233349182	C	T	3.217594	Recessive
18	2763729	T	C	3.161878	Digenic
11	71940602	G	C	2.51846	Compound Het
12	95927820	C	A	2.493677	De Novo
5	148442585	T	C	2.485039	Recessive
2	73115586	A	G	2.481147	Compound Het
9	137005018	A	C	1.579559	De Novo
17	54912344	G	C	0.9798734	Compound Het

CHR	POS	REF	ALT	CADD Score	DDDS Annotation
11	65978677	C	T	35	Heterozygous
12	88519133	C	T	35	Compound Het
12	116413012	C	T	35	Heterozygous
16	56370674	C	T	35	Heterozygous
16	67654615	C	T	35	Heterozygous
16	67654646	C	T	35	Heterozygous
19	52715979	C	T	35	Heterozygous
2	170022537	C	T	35	Compound Het
2	241724480	G	A	35	Heterozygous
3	4856205	G	A	35	Heterozygous
3	176755900	C	A	35	Heterozygous
8	141445217	G	A	35	Compound Het
9	2060867	C	T	35	Heterozygous
9	130428485	G	A	35	Heterozygous
12	116408461	G	A	34	Heterozygous
17	29654736	C	T	34	Heterozygous
19	42474691	C	T	34	Heterozygous
2	50733693	G	A	34	Heterozygous
2	121708956	G	A	34	Heterozygous
2	200173680	C	T	34	Heterozygous
2	200193611	C	T	34	Heterozygous
2	200213431	C	A	34	Heterozygous
2	220432804	G	A	34	Compound Het
2	223086091	G	A	34	Heterozygous
2	240036937	G	A	34	Heterozygous
20	62071037	C	T	34	Heterozygous
20	62071037	C	T	34	Heterozygous
3	4687362	C	T	34	Heterozygous
5	14397222	C	T	34	Heterozygous
5	74722257	G	A	34	Heterozygous
5	177035995	C	T	34	Compound Het
8	61732609	G	A	34	Heterozygous
9	2058457	G	A	34	Heterozygous
9	2060868	G	A	34	Heterozygous
9	2181575	G	A	34	Heterozygous
9	130982480	C	T	34	Heterozygous
1	7797375	C	T	33	Heterozygous
1	220364620	C	T	33	Compound Het
10	123260357	G	A	33	Heterozygous
12	112926887	G	A	33	Heterozygous
13	101756915	G	A	33	Compound Het
16	56545126	C	T	33	Compound Het
2	200193603	C	T	33	Heterozygous
5	177034310	C	T	33	Compound Het
6	42975003	G	A	33	Heterozygous
7	140453133	T	A	33	Heterozygous
9	130444768	G	T	33	Heterozygous
1	220375711	C	G	32	Compound Het
10	76789623	G	A	32	Heterozygous
10	89475544	G	A	32	Compound Het
19	52715971	C	T	32	Heterozygous
2	16085972	G	A	32	Heterozygous
2	74058095	C	T	32	Compound Het
7	140476811	C	T	32	Heterozygous
8	141321437	G	A	31	Compound Het
9	131388821	C	G	31	Heterozygous
9	130984491	A	T	30	Heterozygous
2	166243484	T	A	29.5	Heterozygous
15	93499821	C	T	29.1	Heterozygous
21	38865403	T	C	29.1	Heterozygous

CHR	POS	REF	ALT	Eigen Score	DDDS Annotation
3	71026825	G	A	26.38641	Heterozygous
9	130428485	G	A	18.66891	Heterozygous
21	38862672	A	T	17.87023	Heterozygous
12	52200143	G	A	17.41804	Heterozygous
9	130444768	G	T	17.1107	Heterozygous
15	93499821	C	T	17.07769	Heterozygous
2	16085972	G	A	16.90222	Heterozygous
8	61732609	G	A	16.59939	Heterozygous
21	38865403	T	C	16.35481	Heterozygous
9	130984491	A	T	15.85813	Heterozygous
2	166243484	T	A	15.76658	Heterozygous
3	176755900	C	A	15.47611	Heterozygous
9	2181575	G	A	15.1467	Heterozygous
16	3779563	G	C	15.10561	Heterozygous
5	176720974	G	A	15.02503	Heterozygous
10	50691439	G	A	14.98122	Compound Het
2	223086091	G	A	14.95826	Heterozygous
10	89475544	G	A	14.46367	Compound Het
12	112926887	G	A	14.44845	Heterozygous
16	56370674	C	T	14.15808	Heterozygous
9	2060868	G	A	14.11433	Heterozygous
2	166245954	G	A	13.82436	Heterozygous
5	37017185	G	C	13.72657	Heterozygous
3	4856205	G	A	13.64345	Heterozygous
2	60773293	G	T	13.60111	Heterozygous
2	166848020	A	C	13.58939	Heterozygous
16	56545126	C	T	13.44087	Compound Het
10	123260357	G	A	13.19086	Heterozygous
12	116408461	G	A	12.82125	Heterozygous
6	88239290	A	T	12.55345	Compound Het
21	38858872	T	C	12.37166	Heterozygous
15	26806242	A	G	11.84007	Heterozygous
6	42975013	C	G	11.81166	Heterozygous
16	67654646	C	T	11.75609	Heterozygous
5	74722257	G	A	11.70096	Heterozygous
2	60773348	C	A	11.62838	Heterozygous
16	67645953	A	C	11.627	Heterozygous
2	50733693	G	A	11.39414	Heterozygous
3	176750853	T	C	11.381	Heterozygous
19	42474691	C	T	11.34645	Heterozygous
12	22061091	C	T	11.31592	Heterozygous
9	2060867	C	T	11.24876	Heterozygous
7	148523618	G	A	10.95289	Heterozygous
9	131388821	C	G	10.94621	Heterozygous
2	200193611	C	T	10.82667	Heterozygous
13	101756915	G	A	10.64625	Compound Het
16	67654615	C	T	10.54969	Heterozygous
2	74071966	A	G	10.51547	Compound Het
20	62071037	C	T	10.21207	Heterozygous
20	62071037	C	T	10.21207	Heterozygous
2	60773352	T	G	10.13698	Heterozygous
2	121708956	G	A	10.00757	Heterozygous
2	220432804	G	A	9.959915	Compound Het
2	200213431	C	A	9.895372	Heterozygous
15	40707168	G	A	9.845767	Homozygous
2	200173680	C	T	9.416273	Heterozygous
19	52715979	C	T	9.314533	Heterozygous
7	66098307	A	G	9.302805	Compound Het
9	2058457	G	A	9.161894	Heterozygous
2	166165214	T	G	9.115377	Heterozygous

6	42975013	C	G	29.1	Heterozygous
12	22061091	C	T	29	Heterozygous
12	112888168	T	G	28.7	Heterozygous
4	122765152	T	C	28.6	Compound Het
5	14397221	C	A	28.6	Heterozygous
6	33400583	G	A	28.6	Heterozygous
6	88239290	A	T	28.6	Compound Het
5	177035541	G	A	28.5	Compound Het
16	56309901	T	G	28.4	Heterozygous
2	166245954	G	A	28.3	Heterozygous
7	148523618	G	A	28.3	Heterozygous
3	71026825	G	A	28.2	Heterozygous
9	37783990	T	G	28.1	Homozygous
1	22200454	G	T	28	Compound Het
19	42489099	C	T	28	Heterozygous
10	50691439	G	A	27.9	Compound Het
20	62069997	G	A	27.9	Heterozygous
21	38862672	A	T	27.7	Heterozygous
5	139494382	A	T	27.7	Heterozygous
15	26806242	A	G	27.5	Heterozygous
5	176720974	G	A	27.5	Heterozygous
9	98241382	A	C	27.5	Heterozygous
21	38858872	T	C	27.4	Heterozygous
12	52200143	G	A	27.3	Heterozygous
8	38111197	A	G	27.2	Compound Het
5	37017185	G	C	26.9	Heterozygous
6	157454286	G	T	26.8	Heterozygous
2	220431609	C	G	26.7	Compound Het
15	40707168	G	A	26.5	Homozygous
7	121733190	G	A	26.5	Homozygous
12	13720098	C	G	26.4	Heterozygous
12	52082570	A	G	26.3	Heterozygous
2	166210714	T	C	26.3	Heterozygous
2	166848020	A	C	26.2	Heterozygous
7	66098307	A	G	26.1	Compound Het
2	166165214	T	G	25.9	Heterozygous
10	76789597	G	C	25.6	Heterozygous
11	88911734	C	A	25.5	Compound Het
2	60773293	G	T	25.5	Heterozygous
9	130985129	C	G	25.5	Heterozygous
3	4687356	A	G	25.4	Heterozygous
3	176750853	T	C	25.4	Heterozygous
11	64428503	C	T	25.3	Heterozygous
18	42531866	C	G	25.3	Heterozygous
2	60773352	T	G	25.3	Heterozygous
16	3779449	G	A	25.1	Heterozygous
11	88961072	C	A	25	Compound Het
11	71148919	T	C	24.9	Compound Het
14	89338702	A	G	24.9	Homozygous
8	38111143	A	G	24.8	Compound Het
12	88500654	G	A	24.7	Compound Het
20	62073782	C	T	24.7	Heterozygous
17	36895854	G	A	24.6	Heterozygous
16	67645953	A	C	24.5	Heterozygous
2	60773348	C	A	24.4	Heterozygous
1	147380372	T	G	24.3	Heterozygous
19	11132405	G	A	24.3	Heterozygous
20	62071032	G	C	24.3	Heterozygous
20	62071032	G	C	24.3	Heterozygous
1	236966848	G	A	24.2	Homozygous
18	67684696	A	G	24.1	Compound Het

9	37783990	T	G	9.110487	Homozygous
17	29654736	C	T	8.909366	Heterozygous
12	13720098	C	G	8.899946	Heterozygous
18	48604676	A	G	8.874206	Heterozygous
12	112888168	T	G	8.768866	Heterozygous
18	42531866	C	G	8.763691	Heterozygous
12	52082570	A	G	8.711462	Heterozygous
6	42975003	G	A	8.624231	Heterozygous
16	56309901	T	G	8.553286	Heterozygous
2	241724480	G	A	8.251312	Heterozygous
19	52715971	C	T	8.192446	Heterozygous
3	4687356	A	G	8.085485	Heterozygous
4	122765152	T	C	8.075693	Compound Het
5	14397221	C	A	7.969249	Heterozygous
5	139494382	A	T	7.82685	Heterozygous
8	38111197	A	G	7.805243	Compound Het
9	98241382	A	C	7.779656	Heterozygous
7	121733190	G	A	7.693551	Homozygous
5	177035541	G	A	7.581949	Compound Het
2	166210714	T	C	7.381487	Heterozygous
10	76789597	G	C	7.301231	Heterozygous
10	76789623	G	A	7.20571	Heterozygous
2	170068598	C	T	7.125147	Compound Het
8	141321437	G	A	7.045001	Compound Het
12	49420264	A	C	7.029356	Heterozygous
11	88911734	C	A	6.920876	Compound Het
1	220364620	C	T	6.826655	Compound Het
9	130985129	C	G	6.763433	Heterozygous
11	65978677	C	T	6.471583	Heterozygous
2	200193603	C	T	6.465788	Heterozygous
1	22205601	T	C	6.444776	Compound Het
20	62069997	G	A	6.430767	Heterozygous
9	130982480	C	T	6.384649	Heterozygous
12	116413012	C	T	6.189288	Heterozygous
3	181430372	T	C	6.05597	Heterozygous
19	42489099	C	T	6.037605	Heterozygous
16	3779449	G	A	5.953393	Heterozygous
12	88519133	C	T	5.930911	Compound Het
5	177035995	C	T	5.880149	Compound Het
6	33400583	G	A	5.86361	Heterozygous
2	240036937	G	A	5.733785	Heterozygous
5	177034310	C	T	5.717403	Compound Het
6	157454286	G	T	5.676673	Heterozygous
1	27089742	G	A	5.611343	Heterozygous
8	38111143	A	G	5.516257	Compound Het
8	141445217	G	A	5.406421	Compound Het
5	14397222	C	T	5.331937	Heterozygous
1	147380372	T	G	5.221096	Heterozygous
7	140476811	C	T	5.208471	Heterozygous
20	62071032	G	C	5.201166	Heterozygous
20	62071032	G	C	5.201166	Heterozygous
2	170022537	C	T	4.961519	Compound Het
1	7797375	C	T	4.810332	Heterozygous
3	4687362	C	T	4.79923	Heterozygous
1	220375711	C	G	4.724904	Compound Het
7	148504770	C	T	4.69402	Heterozygous
2	220431609	C	G	4.68659	Compound Het
11	71148919	T	C	4.666896	Compound Het
8	144990697	T	A	4.353859	Compound Het
14	89338702	A	G	4.220837	Homozygous
7	140453133	T	A	4.076613	Heterozygous

7	148504770	C	T	24.1	Heterozygous
15	49060434	T	C	24	Compound Het
2	170068598	C	T	23.7	Compound Het
20	62073787	G	A	23.5	Heterozygous
16	3779563	G	C	23.4	Heterozygous
2	73747141	A	C	23.4	Compound Het
2	74071966	A	G	23.4	Compound Het
15	48738953	T	C	23.3	Heterozygous
8	144990697	T	A	23.3	Compound Het
3	181430372	T	C	23.2	Heterozygous
15	49033814	G	C	23.1	Compound Het
18	48604676	A	G	23	Heterozygous
1	27089742	G	A	22.8	Heterozygous
14	36987163	G	C	22.8	Heterozygous
14	21871618	T	G	21.8	Heterozygous
1	22205601	T	C	21.3	Compound Het
16	88871972	G	A	21.1	Compound Het
16	30718647	C	G	19.3	Heterozygous
10	89473067	G	C	19.16	Compound Het
18	43447589	A	G	18.85	Homozygous
19	7592776	G	A	17.79	Compound Het
19	7592776	G	A	17.79	Compound Het
12	49445799	G	A	17.44	Heterozygous
12	49420264	A	C	16.04	Heterozygous
1	103427786	A	T	15.92	Heterozygous
4	126373642	G	A	15.92	Compound Het
2	170103472	G	A	15.16	Compound Het
17	78172347	C	T	14.22	Heterozygous
11	121008311	G	C	13.73	Compound Het
2	170088242	G	A	11.88	Compound Het
16	89825107	G	C	11.05	Compound Het
2	73675690	A	G	10.95	Compound Het
1	152284382	C	T	10.72	Compound Het
15	49037112	G	C	7.594	Compound Het
3	119133903	G	A	7.204	Heterozygous
4	15602948	T	C	3.997	Homozygous
8	144996236	C	G	1.917	Compound Het
1	52863470	C	A	0.588	Homozygous
10	50678615	T	C	0.002	Compound Het
4	126240408	A	G	0.001	Compound Het

18	67684696	A	G	3.981312	Compound Het
16	30718647	C	G	3.979385	Heterozygous
2	170103472	G	A	3.75835	Compound Het
12	88500654	G	A	3.688746	Compound Het
15	48738953	T	C	3.632492	Heterozygous
10	89473067	G	C	3.419988	Compound Het
11	64428503	C	T	3.203096	Heterozygous
20	62073787	G	A	3.030967	Heterozygous
14	21871618	T	G	2.929387	Heterozygous
2	74058095	C	T	2.851546	Compound Het
17	36895854	G	A	2.815708	Heterozygous
20	62073782	C	T	2.692225	Heterozygous
11	88961072	C	A	2.691227	Compound Het
1	22200454	G	T	2.529277	Compound Het
15	49033814	G	C	2.503511	Compound Het
11	121008311	G	C	2.494084	Compound Het
1	236966848	G	A	2.434117	Homozygous
4	126373642	G	A	2.427697	Compound Het
19	11132405	G	A	2.243166	Heterozygous
18	43447589	A	G	2.231248	Homozygous
14	36987163	G	C	2.226815	Heterozygous
12	49445799	G	A	2.109387	Heterozygous
16	88871972	G	A	2.033848	Compound Het
15	49060434	T	C	1.780718	Compound Het
17	78172347	C	T	1.341495	Heterozygous
16	89825107	G	C	1.309794	Compound Het
3	119133903	G	A	1.197322	Heterozygous
2	73675690	A	G	1.141729	Compound Het
2	73747141	A	C	1.125133	Compound Het
1	103427786	A	T	1.121961	Heterozygous
15	49037112	G	C	0.8937323	Compound Het
4	15602948	T	C	0.8687333	Homozygous
19	7592776	G	A	0.8291489	Compound Het
19	7592776	G	A	0.8291489	Compound Het
2	170088242	G	A	0.6628119	Compound Het
4	126240408	A	G	0.444137	Compound Het
8	144996236	C	G	0.4356646	Compound Het
1	152284382	C	T	0.2425007	Compound Het
1	52863470	C	A	0.1014536	Homozygous
10	50678615	T	C	0.01911332	Compound Het

Supplementary Table 5. CADD and Eigen phred scores for CMG and DDDS variants.
CADD and Eigen scores were ranked from highest to lowest. Heterozygous or dominant-acting variants are highlighted in grey.

	All Mutations	Mutations in cancer predisposition genes	Mutations in all other genes
Total	57850	5429	52421
# Pathogenic	56559	5326	51233
# Benign	1291	103	1188
% Pathogenic	97.8%	98.1%	97.7%

Supplementary Table 6. MAPPIN pathogenicity predictions for all HGMD disease-causing mutations. The pathogenicity predictions are split into mutations that occur in cancer predisposition genes vs. all other genes.
